# Lycorine (*Lycoris radiata*)—a unique natural medicine on breast cancer

**DOI:** 10.1111/jcmm.70032

**Published:** 2024-08-22

**Authors:** Qinbing Xue, Bing Wang, Jie Feng, Chaoyu Li, Miao Yu, Yan Zhao, Zheng Qi

**Affiliations:** ^1^ Engineering Research Center for Medicine, Ministry of Education Harbin University of Commerce Harbin China; ^2^ School of Food Engineering Harbin University of Commerce Harbin China; ^3^ Department of Medical Imaging The Fourth Affiliated Hospital of Harbin Medical University Harbin China

**Keywords:** breast cancer, cell cycle arrest, lycorine, mitosis, M‐phase

## Abstract

Breast cancer (BC) is one of the most common types of cancer among women worldwide. Lycorine (*Lycoris radiata*), a small molecule derived from the traditional Chinese herb *Amaryllidaceae* plants, has appeared potential effect on inhibiting the growth of cancer cells and inducing apoptosis in various types of cancer with minor side effects. To discuss the therapeutic effects and molecular mechanisms of lycorine on BC established by lycorine‐treated S180 tumour‐bearing mice in vivo. Furthermore, both the mitotic and microtubule assembly dynamics genes were performed by qPCR assays, and the protein expression associated with mitotic arrest was investigated by western blot. Lycorine was demonstrated to reduce sarcoma growth of S180 tumour‐bearing mice and inhibit the proliferation of MCF‐7 cells in concentration‐dependent manner. Moreover, lycorine induced M phase cell cycle arrest via interfering with the mitotic apparatus regulated the expression of 20 genes and 15 proteins in cell cycle progression. Furthermore, this study confirmed that the potential effect of lycorine on BC might be mediated by cell cycle arrest in M phase for the first time. These results would be the consequence of exploitation of lycorine as a potential drug for BC therapy, however further preclinical and clinical studies are still needed.

## 1 INTRODUCTION

Cancer is the leading causes of death worldwide, by 2040, the number of new cases is expected to be 29.5 million and the number of cancer‐related deaths up to 16.4 million per year. Among these, breast cancer (BC) has surpassed lung cancer as the most common cancer in women, according to 12.5% of all new annual cancer cases in 2022.^1^ Women at increased risk of BC have several options to reduce, including surgery, lifestyle options and medication. Still, the therapeutic strategies against BC mainly rely on resection and chemotherapy, on the other hand, the clinic treatment owns high risk of adverse side effects.^2^

Traditional Chinese medicine (TCM) has garnered more attention for its definite curative effect and low toxicity.^3^, ^4^ TCM has a long history of application on BC targeted therapies with growing popularity as it promotes postoperative physical recovery, reduce surgical complication rate, diminish the risk of recurrence and metastasis, attenuate chemotherapy toxicity, and reverse drug resistance.^5^ Lycorine is a bioactive phenanthridine alkaloid extracted from the bulbs of *Lycoris radiata* (family *Amaryllidaceae*), which possess effects of anti‐cancer, antibacterial, anti‐inflammatory, enzyme inhibitory and analgesic effects.^6^, ^7^ Moreover, aberrancy in the cell cycle progression is the key mechanism underlying tumorigenesis, thereby, anticancer therapeutic targets mainly depend on the regulating cell cycle progression.^8^ However, the mechanisms underlying the therapeutic effects of lycorine on BC via cell cycle arrest are still unclear.

To data, cell cycle progression is mainly focused on replication of both the genomic DNA and its subsequent segregation, where they occur in eukaryotic cells during the distinct cell cycle phases. Most of the cancer cells are confirmed to undergo uncontrolled cell cycle progression, thereby, cell cycle checkpoints need to be defective for a cell to become cancerous.^9^ This appeared to be consistent with the previous study; it indicated that the cancer cells are compromised to arrest the cell cycle instead of undergoing uncontrolled cell division.^10^ Obviously, cell cycle arrest is essential for the cancer cell viability; in this perspective, the differences between DNA damage check point and replication stress checkpoint responses are essential in the context of cancer. Analogously, the cancer cells strongly rely on the functional mitotic checkpoints to prevent catastrophic chromosome mis‐segregation.^11^ Taken together, cell cycle arrest phase could represent as an “Achilles' heel” of cancer, which are crucial for the cell cycle control and the new therapeutic opportunities revealing.

To our knowledge, few studies have focused on the therapeutic effects of lycorine on BC by cell cycle progression. Thus, this study aims to (1) evaluate the anti‐carcinoma cells effect of lycorine in vivo; (2) compare the inhibiting effect of lycorine on MCF‐7, HepG2, MDA‐MB‐231, A549 and SGC‐7901 cells; (3) confirm the cell cycle arrest of lycorine on BC by morphological observation (4) and explore the mechanisms in both the relative expression of genes and proteins. This should be the consequence of lycorine exploitation as a potential drug for BC therapy.

## 2 MATERIALS AND METHODS

### 2.1 Chemicals and reagents

The human breast cancer cell line MCF‐7, hepatoma cell line HepG2, breast carcinoma cell line MDA‐MB‐231, gastric carcinoma cell line SGC‐7901 and non‐small cell lung cancer cell line A549 were cultured and maintained at the Postdoctoral Research Workstation of the Engineering Research Center for Medicine, Harbin University of Commerce (Harbin, China). These carcinoma cell lines were preserved in cryogenic storage and propagated for fewer than 50 generations in laboratory. The Short Tandem Repeat (STR) was checked on National Infrastructure of Cell Line Resource website (Figure S1). Lycorine was purchased from Aladdin Biotechnology (molecular formula: C_16_H_17_NO_4_; purity >98%, China, Figure S2). Cyclophosphamide (CTX) and vincristine (VCR) were obtained from Yuanye Biotechnology (Shanghai, China). Fetal bovine serum (FBS) was purchased from Corning Life Science, New Zealand. RPMI‐1640 medium and Dulbecco's Modified Eagle Medium (DMEM) were obtained from Gibco (USA). The PageRuler Prest Protein Ladder was supplied by Fermentas Biotechnology (USA). Additionally, 3‐(4,5‐dimethylthiazol‐2‐yl)‐2,5‐diphenyltetrazolium bromide (MTT), phenylmethanesulfonyl fluoride (PMSF), western and cell lysis buffer IP, BCA Protein Assay Kit were sourced from Beyotime Biotechnology (Jiangsu, China). The DAB Concentrated Kit was obtained from Zhong Shan‐Golden Bridge Biological Technology (Beijing, China). Primary antibodies targeting CyclinB1, CDK1, p21, Aurora A, Aurora B, p‐H3, PLK1, BRCA1, BUBR1, STAT3, PAK1, CAMK4, PKA, ERK1/2, GAPDH and β‐actin were purchased from Bioss Biotechnology (Beijing, China). The secondary antibodies included horseradish peroxidase (HRP)‐conjugated anti‐mouse or anti‐rabbit, and FITC‐labelled goat anti‐mouse antibodies obtained from Beyotime Biotechnology (Jiangsu, China). All reagents employed in this study were of analytical grade, and the comprehensive list of instruments and reagents used are detailed in Tables S1 and S2.

### 2.2 Animals and design

A total of 60 Kunming mice (clean grade, half male and half female, 4 weeks old and weighing 20 ± 2 g) were procured from Liaoning Changsheng Biotechnology Co. Ltd. (licence number: SCXK (Liao) 2010‐0001), and maintained in a temperature‐controlled room with 12 h light/dark cycle and free access to the given food and water. All experimental protocols were in strict compliance with the guidelines approved by the Animal Care and Use Committee of Harbin University of Commerce, ensuring adherence to animal ethics and welfare standards (Figure S3). The S180 tumour cells were obtained from the China Center for Type Culture Collection. After an initial adaptation period of 1 week, the mice were injected subcutaneously on their right flank with the S180 cell line (2 × 10^6^ cells/mL). When the tumour diameters reached approximately 1 cm after 7 days, the mice were systematically divided into six groups (*n* = 10, half male and half female in each group): control, model (saline), lycorine at low (10 mg/kg·bw), middle (20 mg/kg·bw) and high doses (40 mg/kg·bw), and CTX (2 mg/kg·bw). Each group received a consistent daily dose of 0.2 mL via intraperitoneal injection for a duration of 7 days. The S180 tumour‐bearing mice were euthanized 24 h after the final administration. The tumours, thymuses and spleens were removed and weighed to calculate tumour weight, thymus index and spleen index. The tumour growth inhibition rate (IR) was calculated by the formula IR (%) = (1−TWt/TWm) × 100%, where TWt is the mean value of tumour weight after lycorine and CTX treatment, and TWm is the mean value of tumour weight of model group,^12^ spleen or thymus index (mg/g) = the weight of spleen or thymus (mg)/body weight (g).

### 2.3 Cell viability assay

MCF‐7, HepG2, SGC‐7901, A549 and MDA‐MB‐231 cells were cultured in 96‐well plates with 10% foetal bovine serum and various concentrations of lycorine (2, 4, 8, 16, 32 and 64 μmol/L) for 24 h (*n* = 6). Cells were cultured at a density of 5 × 10^4^ cells/well.^13^ Then, 50 μL of 0.5 mg/mL MTT solution was added to each well and incubated at 37°C in a humidified incubator with 5% CO_2_ for 4 h. After incubation, the supernatant was discarded and 150 μL of DMSO was added into each well. Finally, the absorbance values (OD values) of cells were read at 490 nm using a microplate reader for cell viability measurements.

### 2.4 Immunofluorescent and cell cycle progression detection

MCF‐7 cells were treated with lycorine (7, 14 and 28 μmol/L) for 48 h, and VCR (0.625 μmol/L) was used as positive control intervention. The cells were washed three times with PBS solution, and afterwards the cells were stored in 75% ethanol at −20°C overnight. Then, the fixed MCF‐7 cells were stained in cold PI solution in the dark for 30 min. The OD values were read by a flow cytometer at an excitation wavelength of 488 nm, furthermore, the flow cytometric data were obtained using the Beckman‐Coulter EPICS XL‐MCL flow cytometer.

4% paraformaldehyde was used to fix MCF‐7 α‐tubulin cells in 6‐well culture plates for 10 min. After that, cells were membrane permeabilized in 0.1% Triton X‐100 for 5 min. In addition, 5% BSA blocking buffer was used to block the cells. Overnight at 4°C, cells were treated with a primary antibody against tubulin (1:1000). Then, the cells were washed in PBS solution twice. The sections were then treated with anti‐goat IgG secondary antibody (1:3000) for 1.5 h. Finally, fluorescence microscope was then used to observe tubulin FITC stained MCF‐7 cells.

### 2.5 Total RNA extraction and gene expression analysis

A total of 101 cell cycle‐related target genes were detected and the primer sets are listed in Table S4. Among them, two DNA damage related gene (*RAD51* and *MRE11A*), five signal transduction related gene (*STAT3*, *ERK*, *PKA*, *CAMK4* and *PAK1*), six microtubule‐related gene (*ICIS*, *KIF2C*, *STMN1*, *KATNA1*, *MAP2* and *MAPT*) and seven M‐phase related gene (*AURKB*, *CDC2*, *CCNB2*, *CCNF*, *CDC25C*, *CDC6* and *CDC20*) with high expression change were selected, which quantified in duplicate using Step One Plus™ Real‐Time PCR system (Wcgene Biotech, Inc., Shanghai, China). The specificity of the qPCR products was also verified by melting curve analysis in Figure S4, and the relative abundance of cycle arrest genes was calculated on the basis of 2^−ΔΔ*C*t^ method, where ΔΔ*C*_t_ = Δ*C*_t_ (target gene)−Δ*C*_t_ (internal reference gene).^14^

### 2.6 Protein expression detection

The protein bands (CyclinB1, CDK1, p21, Aurora A, Aurora B, STAT3, PAK1, CAMK4, PKA and ERK1/2) were quantified by Image‐pro plus 6.0 imaged using hypersensitive luminescence solution and the densitometry analysis. Subsequently, after FITC staining, 400‐mesh nylon net was used to filter the cells. The relative fluorescence intensity of FITC‐stained protein (p‐H3, PLK1, BRCA1 and BUBR1) detected with flow cytometry was analysed.

### 2.7 Statistical analysis

All experiments were repeated at least three times. Statistical analyses were performed using SPSS 15.0 statistical software, and the data were presented as mean ± standard deviation (SD). The Dunnett and Tukey test were performed to compare between the treatment groups and the control groups. Heatmap (Java Treeview 1.1.6) and Cluster 3.0 were used to analyse the correlation between proteins and genes expression, and *p* < 0.05 was considered statistical difference, *p* < 0.01 presented significant difference, and *p* < 0.001 presented extremely significant difference.

## 3 RESULTS

### 3.1 Effects of lycorine on tumour weight, spleen index and thymus index in vivo

**FIGURE 1:**
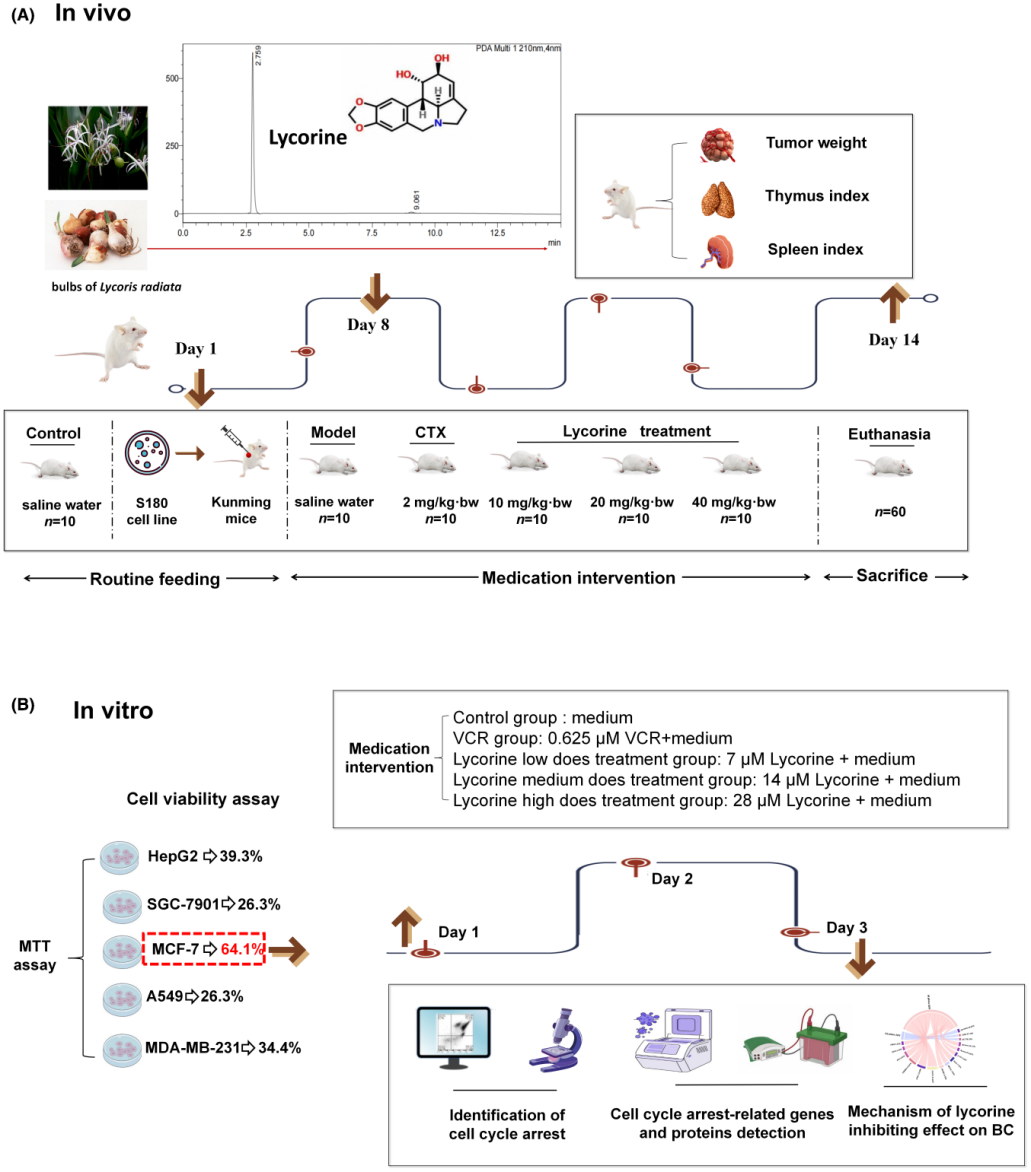
The schedule of experimental design. (A) Study design of animal experiment and treatment in vivo. (B) Study design of cell experiment in vitro.

**FIGURE 2:**
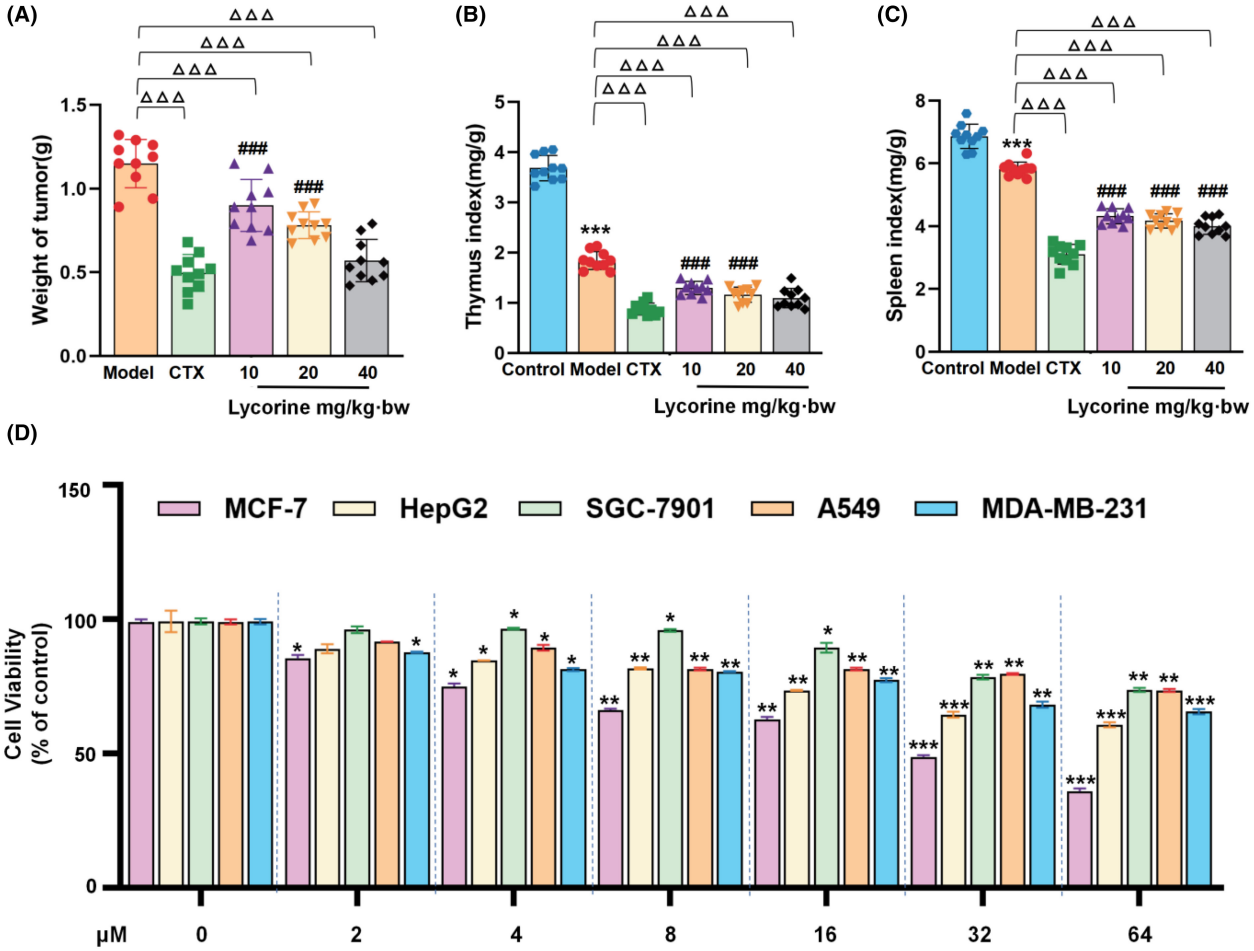
Lycorine inhibited S180 tumour growth and stimulated the host immune response. (A) Tumour weights of mice in each group (*n* = 10). (B) Thymus indexes of mice in each group (*n* = 10). (C) Spleen indexes of mice in each group (*n* = 10). (D) The cell viability variation of MCF‐7, HepG2, SGC‐7901, A549 and MDA‐MB‐231 cells treated with lycorine of 2, 4, 8, 16, 32 and 64 μmol/L (*n* = 6). Data were expressed as mean ± standard deviation. Significant difference was indicated as ^###^ *p* < 0.001 presented extremely significant difference compared with the CTX group. *p < 0.05, **p < 0.01 and ***p < 0.001, compared with the control group. ^△△△^ *p* < 0.001, compared with the model group.

The schedule of animal experiment in vivo and cell assay in vitro was presented in Figure 1. Lycorine dramatically suppressed tumour growth of S180 tumour‐bearing mice and appeared a dose‐dependent manner, the tumour inhibition rates were 21.7% (10 mg/kg·bw of lycorine), 32.1% (20 mg/kg·bw of lycorine) and 50.6% (40 mg/kg·bw of lycorine), respectively (Figure 2A and Table S5). The tumour weight intervened by 40 mg/kg·bw lycorine (0.57 ± 0.13 g) were significantly reduced compared with the model group (*p* < 0.001), and its curative effect of high dose is consistent with that of CTX (0.49 ± 0.08 g). Following treatment with lycorine, the thymus and spleen indexes of S180 tumour‐bearing mice appeared little effect (Figure 2B,C), while there was no significant difference between high dose and low dose groups (*p* = 0.079, *p* = 0.1470, respectively). Additionally, the thymus and spleen indexes showed a significant decreased in CTX groups (0.88 ± 0.65 and 3.11 ± 0.13, respectively) in comparison with high dose groups of lycorine (1.093 ± 0.28 and 4.01 ± 0.53, respectively). These results indicated that lycorine could inhibit the immune function of S180 model mice as well as anti‐tumour effect. Taken together, the aforementioned date indicated that lycorine could inhibit the growth of tumour, likewise, lycorine appeared little effect on the thymus and spleen indexes.

### 3.2 Inhibition of lycorine on MCF‐7, HepG2, MDA‐MB‐231, A549 and SGC‐7901 cells in vitro

The inhibition rate of lycorine in dose of 64 μmol/L on cell viability in vitro was ranked as human breast cancer cell line MCF‐7 (64.1%) > human hepatoma cell HepG2 (39.3%) > human breast carcinoma cell line MDA‐MB‐231 (34.4%) > human gastric carcinoma cell line SGC‐7901 (26.3%) and non‐small cell lung cancer cell line A549 (26.3%) (Figure 2D). The half‐maximal inhibitory concentration (IC_50_) values of lycorine on MCF‐7 cells were 13.98 μmol/L (Table S3). In this way, three different doses (7, 14 and 28 μmol/L) of lycorine were selected for the further experiments.^15^ These results suggested that lycorine was revealed to capable of BC treatment in comparison to the other cancer cells.

### 3.3 Identification and analysis of cell cycle arrest

**FIGURE 3:**
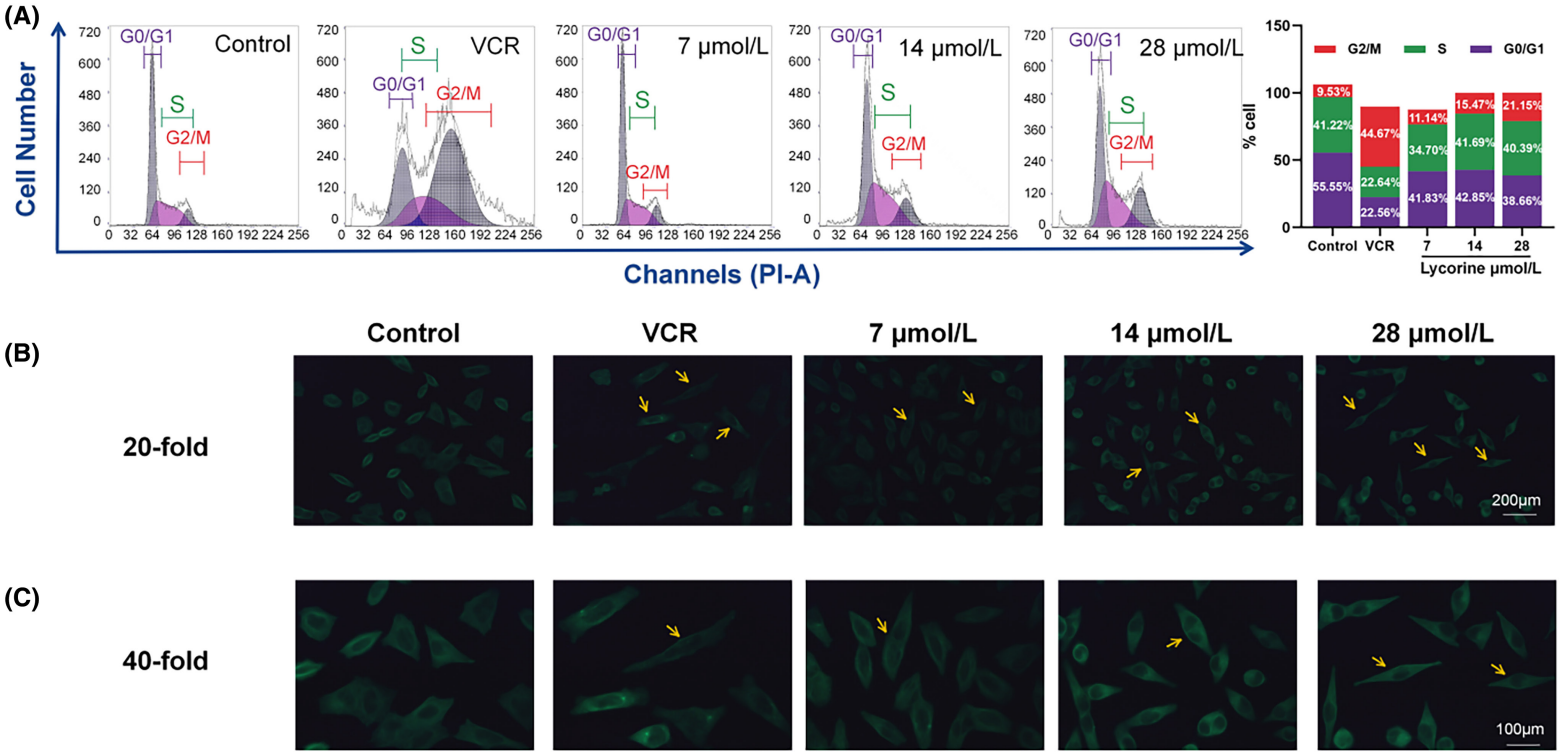
Lycorine induced cell cycle arrest in MCF‐7 cells. (A) Flow cytometric analysis of phases percentage of the cell cycle in MCF‐7 cells treated with lycorine (7, 14 and 28 μmol/L), 0.625 μmol/L VCR for 24 h and stained with PI. (B) ×20, (C) ×40 α‐tubulin changes detected by fluorescence microscopy. Anti α‐tubulin and FITC‐conjugated goat anti‐rabbit IgG (green) were used to label microtubules. Yellow arrows presented abnormal α‐tubulin morphology in MCF‐7 cells.

To validate whether lycorine could inhibit cell proliferation by the effect of inducing cell cycle arrest, the percentage of the cell cycle phases of MCF‐7 cells was analysed after treatment with lycorine. Lycorine induced the accumulation of cells in G2/M‐phase cell‐cycle arrest in a dose‐dependent manner (Figure 3A). The cells proportion of G2/M phase increased with the doses of lycorine from 11.14% (7 μmol/L), 15.47% (14 μmol/L) to 21.15% (28 μmol/L), which appeared significantly increased compared with the control group (9.53%). However, a gap in the curative effect was observed between 28 μmol/L of lycorine (21.15%) and 0.625 μmol/L of VCR (44.67%).^16^ Additionally, proportion of G0/G1 and S phase cells were considered no significant difference. The results demonstrated lycorine induced MCF‐7 cell cycle arrest in G2/M phase arrest.

To investigate the cellular response to lycorine treatment induced G2/M phase cell cycle arrest, the morphological changes of MCF‐7 cells was observed by immunofluorescence analysis (Figure 3B,C). MCF‐7 cells in the control group displayed distinct cell boundaries and uniform shapes, with α‐tubulin uniformly distributed along the cell peripheries. In contrast, cells treated with VCR exhibited indistinct boundaries and an increase in cell volume. Notably, after 48‐h exposure to lycorine, MCF‐7 cells showed significant alterations in tubulin polymerization. These cells manifested disorganized morphology, blurred peripheries, enlargement, irregular shapes and distorted α‐tubulin structures. Similarly, the morphological alterations in cells treated with a high dose of lycorine (28 μmol/L) paralleled those observed in the VCR treatment group. Thus, these findings demonstrated that lycorine induced tubulin polymerization at the cellular and molecular levels, which indicated that lycorine had tubulin‐targeting function in MCF‐7 cells.

### 3.4 Abundance expression of cell cycle‐regulatory genes

**FIGURE 4:**
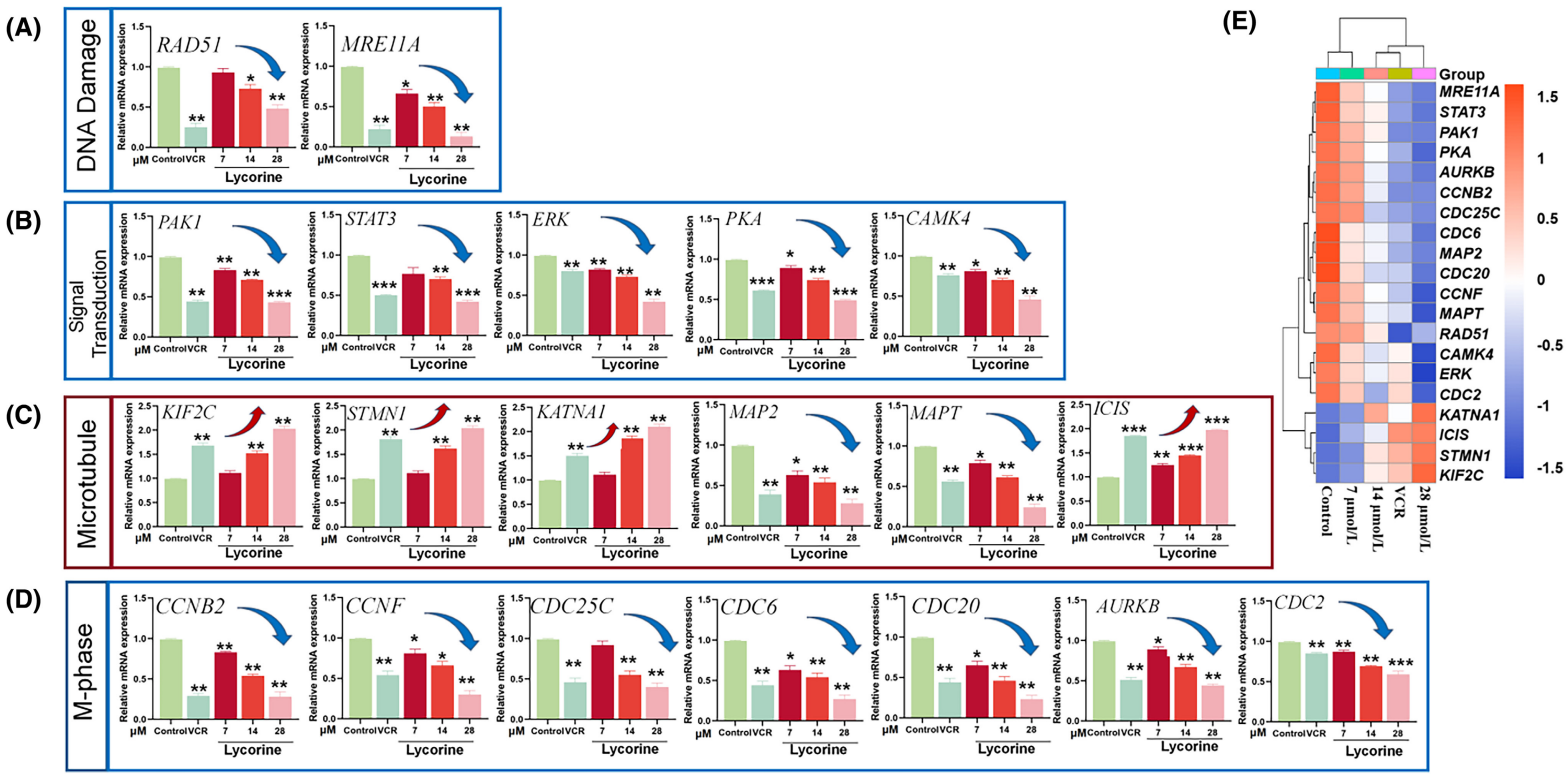
Effect of lycorine treatment on expression of relative genes in MCF‐7 cells. (A) Effect of lycorine on the expression of DNA damage relative genes in MCF‐7 cells (*n* = 3). (B) Effect of lycorine on the expression of signal transduction relative genes in MCF‐7 cells (*n* = 3). (C) Effect of lycorine on the expression of microtubule relative genes in MCF‐7 cells (*n* = 3). (D) Effect of lycorine on the expression of M‐phase relative genes in MCF‐7 cells (*n* = 3). (E) Heatmap of various doses of lycorine treatment on MCF‐7 intracellular cycle‐related gene expression. Significant differences indicated as *p < 0.05, **p < 0.01 and ***p < 0.001 compared with the control group.

To elucidate the underlying mechanisms, the cell cycle‐regulatory genes were examined by qPCR assay. Genetic changes are the primary signal of cellular changes, in this way, all the genes of 101 in cell cycle were detected. Among them, a sub of two genes (*MRE11A* and *RAD51*) down regulated in DNA damage (Figure 4A), five genes down regulated (*STAT3*, *CAMK4*, *PKA*, *PAK1* and *ERK*) in signal transduction (Figure 4B), four genes up regulated (*KATNA1*, *ICIS*, *STMN1* and *KIF2C*) and two genes (*MAPT* and *MAP2*) down regulated in microtubule (Figure 4C), and seven genes down regulated (*CCNB2*, *CCNF*, *CDC25C*, *CDC6*, *CDC20*, *AURKB* and *CDC2*) in M‐phase (Figure 4D). The effect of lycorine on MCF‐7 by regulating the expression of intracellular cycle‐related genes was performed in order to further capture the interactions between 20 genes (Figure 4E). These 20 related genes were divided into four categories, the relative abundance expression of *MRE11A*, *STAT3*, *PAK1*, *PKA*, *AURKB*, *CCNB2* and *CDC25C* were similar; genes *CDC6*, *MAP2*, *CDC20*, *CCNF*, *MAPT* and *RAD51* clustered into one category; the expression of *CAMK4*, *ERK* and *CDC2* were similar; while *KATNA1*, *ICIS*, *STMN1* and *KIF2C* were clustered in one category. Herein, these findings demonstrated that lycorine weakens cell signal transduction, reduce repairability of DNA damage, enhances microtubule dynamics and leads to mitotic arrest in MCF‐7 cells. On the other hand, the expression of genes in control group and low dose of lycorine were presented similar, and high dose of lycorine was revealed to have same effect with VCR group.

### 3.5 Cell cycle arrest‐related protein expression

**FIGURE 5:**
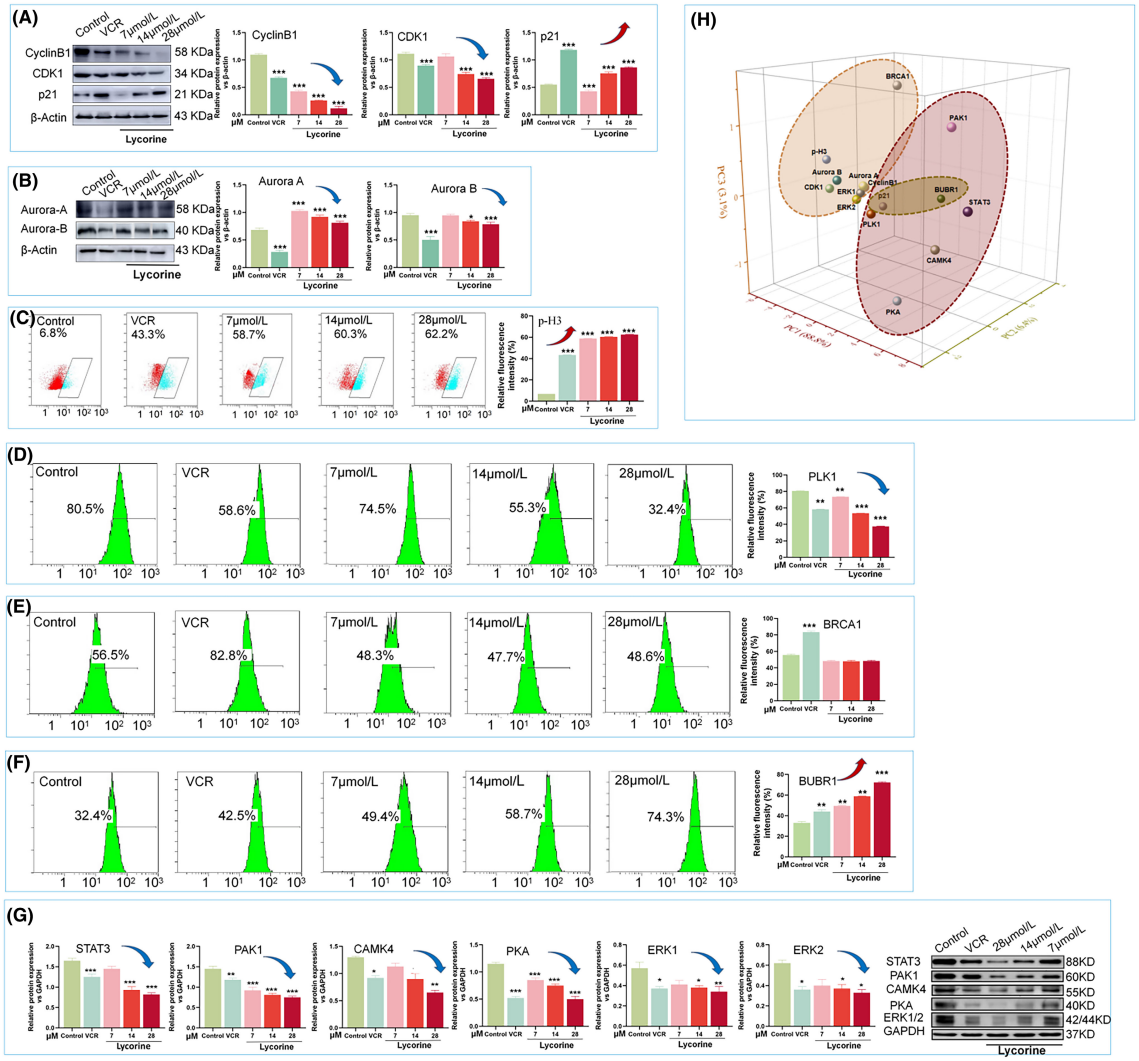
Expression of proteins in cell cycle arrest. (A) Expression of protein CyclinB1, CDK1, p21 by western blot. (B) Expression of protein Aurora A and Aurora B by western blot. (C, D, E and F) Quantification of the p‐H3, PLK1, BRCA1 and BUBR1 protein in cells by FITC staining with flow cytometry. (G)Expression of protein STAT3, PAK1, CAMK4, PKA and ERK by western blot. (H) PCA ordination plot for eigenvalue decomposition covariance matrixes among cell cycle arrest relative proteins based on the relative abundance. Significant differences were indicated as *p < 0.05, **p < 0.01 and ***p < 0.001 compared with the control group.

To further confirm the effect of lycorine on cell cycle arrest‐related protein activity, a total of 15 protein expression was discussed both in M‐phase (Table S6). Specifically, western blot analysis revealed that lycorine enhanced the expression of cell cycle associated marker p21, but decreased the expression of CyclinB1, CDK1, Aurora A, Aurora B, STAT3, CAMK4, PAK1, PKA, ERK1 and ERK2 in MCF‐7 cells after 48 h (Figure 5A,B,G). Furthermore, lycorine also significantly upregulated the expression of mitotic phase marker p‐H3 and BUBR1 (Figure 5C,F) and down‐regulated the expression of PLK1 (Figure 5D), however the expression of BRCA1 had no obvious variation (Figure 5E). Repeat all experiments three times (Figure S5). The results indicated that lycorine triggered the M‐phase cell cycle arrest in MCF‐7 cells.

In order to elucidate the specific involvement of proteins in cell cycle arrest, a principal component analysis (PCA) was conducted. This analysis revealed that among the proteins related to cycle arrest, PLK1, STAT3, PAK1, CAMK4 and PKA were predominantly grouped in the first principal component (PC1), accounting for 88.8% of the variance in this component. On the other hand, p21 and BUBR1 contributed to 6.4% of the variance in the second principal component (PC2), while the remaining proteins were associated with 3.1% in the third principal component (PC3) (Figure 5H and Table S7). These results indicate that the cell cycle arrest in MCF‐7 cells induced by lycorine is, at least in part, a consequence of modulations in the expression of certain cell cycle‐related proteins. Furthermore, the data suggests that the M‐phase cell cycle arrest induced by lycorine in MCF‐7 cells may be mediated through the action of proteins such as PLK1, STAT3, PAK1, CAMK4 and PKA.

### 3.6 Mechanism of lycorine inhibiting effect on breast cancer

**FIGURE 6:**
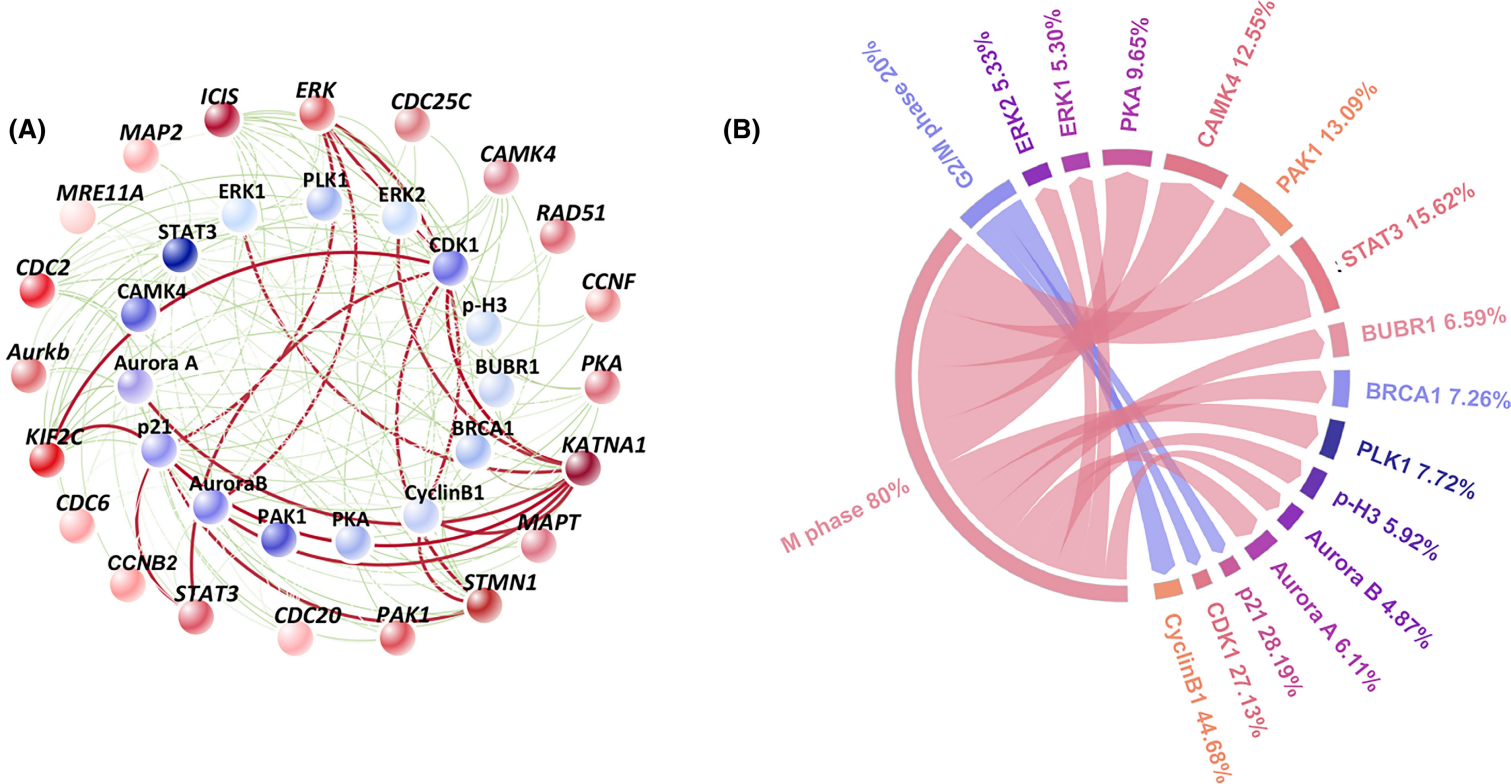
The mechanism of lycorine‐induced cell cycle arrest related. (A) Correlation between cell cycle arrest related proteins and genes (*n* = 3, the darker colour indicated the greater contribution in proteins and genes, the red line represents *p* < 0.001 and the green line represents *p* < 0.05). (B) The contribution of proteins analysed by chord diagram. The size of the connected ribbon indicated by the shared cell cycle and proteins.

In order to evaluate the impact of lycorine on mitotic arrest within MCF‐7 cells, a comprehensive cluster and correlation analysis was conducted focusing on 20 selected genes and 15 associated proteins. The intensity of the colour representation corresponded to the expression levels of genes and proteins, with darker shades indicating higher expression. Likewise, the thickness of the lines in the analysis denoted the strength of the relationship between genes and proteins (Figure 6A and Table S8). A particularly strong correlation (*p* < 0.001) was observed between protein CDK1 and genes *STMN1*, *KIF2C*, *KATNA1*, *STAT3*, *ERK*, protein CyclinB1 and genes *STMN1*, *KATNA1*, *ERK*, protein p21 and genes *STMN1*, *KIF2C*, *KATNA1*, *STAT3*, *ERK* and protein Aurora B and gene KATNA1 and *ERK* in MCF‐7 cells.

By contrast, the cell cycle progression was classified into two types, which were M phase (80%), and G2/M phase (20%). The results indicated that lycorine induced cell cycle arrest mainly in M phase. Meanwhile, the expression of M phase‐related protein ranked as STAT3 (15.62%) > PAK1 (13.09%) > CAMK4 (12.55%) > PKA (9.65%) > PLK1 (7.72%) > BRCA1 (7.26%) > BUBR1 (6.59%) > p‐H3 (5.92%) > ERK2 (5.33%) > ERK1 (5.30%), followed by Aurora B of 4.87%. Cyclin B1, p21 and CDK1 accounted for the fraction of 44.68%, 28.19% and 27.13% in G2/M phase, respectively (Figure 6B). In a world, lycorine significant inhibited MCF‐7 cell cycle arrest in M phase (Figure 6).

## 4 DISCUSSION

BC remains a recalcitrant cancer in women worldwide, thus novel therapeutic strategies of TCM with low side effects are urgently needed. Lycorine, a principal alkaloid derived from *Lycoris radiata* of the *Amaryllidaceae family* has a well‐documented history for its broad pharmacological effects, which including anti‐inflammatory, antiviral and antitumor properties.^17^, ^18^ Notably, lycorine not only has inhibitory effects on tumour growth but also has extensive activity that induces apoptosis. It suppresses constitutive STAT3 activation by up‐regulating the expression of SHP‐1 and down‐regulating the expression of STAT3 target proteins, thus induce apoptosis and cell cycle arrest.^19^ Lycorine has inhibiting effect on BC with less damage to the immune system for the first time. However, more research is needed to fully understand potential therapeutic effect of lycorine as a drug of BC treatment and its safety profile.

Chemotherapy drugs are often associated with immunosuppression as a major adverse effect, while a concern not evident in the case of lycorine. The thymus and spleen pivotal in orchestrating adaptive immune responses play a crucial role in this context.^20^ CTX identified as the most efficacious among over 1000 anti‐tumour compounds in combating 33 cancer types.^21^ In contrast, lycorine not only impedes tumour proliferation but also mitigates the frequency of adverse effects. This dual capability of lycorine marks a significant advancement in cancer therapy in acting as an anti‐tumour agent while concurrently diminishing the likelihood of immunosuppressive consequences.

By contrast, stomach, lung, liver and breast cancers rank among the top malignancies worldwide in terms of both morbidity and mortality.^22^, ^23^, ^24^, ^25^, ^26^ Moreover, the inhibitory effects of lycorine on the growth of MCF‐7, MDA‐MB‐231, SGC‐7901, A549 and HepG‐2 cells were demonstrated by MTT assay in vitro. Lycorine appears obvious inhibitory effects on MCF‐7 cell proliferation in a dose‐dependent manner, and the IC_50_ values of lycorine for MCF‐7 cells was 13.98 μmol/L. Last but not least, the MCF‐7 cells were considered sensitive to lycorine.

Cell cycle arrest is a critical process where the normal progression of the cell cycle is halted that in response to various stimuli such as DNA damage, replication stress and oncogene activation.^27^ This mechanism has been proposed as a viable therapeutic strategy in cancer treatment.^28^ However, the specific pathway through which lycorine induces cell cycle arrest in cancer cells remains to be fully elucidated. Firstly, the results demonstrated lycorine induced MCF‐7 cell cycle to arrest in G2/M phase by flow cytometer. Furthermore, a sub of 101 genes in cell cycle were detected, among them, 20 target genes and 15 cell cycle arrest related proteins with high expression changes were discussed. More importantly, CDK1 and CyclinB1 are critical in G2 phase checkpoint.^19^ It is demonstrated that evodiamine induced M‐phase cell cycle arrest via the p21 signalling pathway in ESCC cells.^29^ The expression of protein p21 was up‐regulated by lycorine treatment in this study as well. p21 is a cell cycle protein kinase inhibitor that can bind and inhibit various cell cycle protein kinases, then finally leads to cell cycle arrest. The expression of p21 upregulates after DNA damage, thereby preventing cells from entering the M phase. The expression of protein CDK1 down‐regulates by p21, which causes the down regulation of CyclinB1 further inhibited the activity of CDK1 in MCF‐7 cells.

The phosphorylation of histone H3 (p‐H3) is a critical step in the regulation of the cell cycle, playing a key role in the regulation of DNA replication and chromosome segregation.^30^ During the cell cycle, an increased in expression of p‐H3 may indicate that lycorine induced mitotic arrest in MCF‐7 cells. BUBR1 is a key protein in mitotic checkpoint, which plays a role in maintaining checkpoint activation in chromosome kinetochore and cytoplasm, and its expression peaks at G2/M phase with the progress of cell cycle. The upregulation of BUBR1 indicates active cellular responses to issues in chromosome segregation or spindle attachment. This study revealed that lycorine treatment significantly enhanced the mitotic phase marker protein p‐H3, p21 and BUBR1 expression, indicating that lycorine triggered the M‐phase cell‐cycle arrest in MCF‐7 cells, instead of the G2 phase. In addition, it suggested that lycorine induced M‐phase arrest may be mediated by p21.

The phosphorylation of p‐H3 is catalysed by mitotic kinases such as Aurora A and Aurora B, which recognize and bind to the p‐H3 modified histones. Any disruptions in p‐H3 phosphorylation levels may lead to a variety of chromosomal abnormalities and contribute to the development of diseases such as cancer.^31^ In this research, a notable down‐regulation in the expressions of Aurora A and Aurora B in response to lycorine treatment. This down‐regulation signifies a substantial impact on these critical mitotic regulators, underscoring the potential of lycorine as a modulator of key cellular processes. Aurora kinases play an essential role are crucial for the accurate progression of mitosis in various aspects of cell division, including spindle assembly, chromosome alignment and segregation, and their activity and stability. Likewise, PLK1 was the key checkpoint to recovery from mitotic arrest mediated by the DNA damage after DNA damage repair.^32^ The decrease in PLK1 expression represents that DNA damage response in MCF‐7 cells after lycorine treatment.

*AURKB*, *CCNB2*, *CDC6*, *CCNF*, *CDC2*, *CDC20* and *CDC25C* play crucial roles in the cell cycle, particularly during the M phase. Down‐regulation of these genes can lead to M phase arrest and cause cell division hindering. *AURKB* and *CDC2* are key proteins regulating microtubule stability and chromatin condensation. *CCNB2* forms a complex with *CDC2* to drive the cell into M phase. *CDC20* and *CDC25C* play roles in activating *CDC2*, while *CCNF* is involved in the degradation of *CDC6*, affecting the licensing of DNA replication. A decrease in the expression of *RAD51* and *MRE11A* may increase the risk of DNA damage and reduce the cell's ability. *STAT3*, *PAK1*, *CAMK4*, *PKA* and *ERK1/2* are key genes involved in regulating the signal transduction of mitosis in cells. After lycorine treatment, the down‐regulation of these genes may lead to impairment of the signalling pathways by affecting the normal progression of the cell cycle.

Apart from cell‐cycle arrest, triggering microtubules dynamics is another appealing option for cancer treatment.^33^ The microtubule targeting agents used in cancer chemotherapy work by disrupting the normal structure and stability of microtubules, leading to cell cycle arrest and apoptosis. In the control group, the microfilaments and microtubules appeared as bundles, and the cell morphology was normal. With the increasing concentration of lycorine, the bundle‐like fibres of microtubule proteins gradually elongated. In the high‐dose group, the microtubule morphology was similar to that in the VCR group. This result suggests that lycorine may act on microtubules and be related to the dynamic equilibrium of microtubules. Moreover, disrupting the dynamic balance of microtubule depolymerization and polymerization is a potential target for the development and research of antitumor drugs. *KATNA1*, *STMN1* and *KIF2C* are key proteins involved in microtubule dynamics, with implications in cancer progression and potential targets for anticancer drug development.^34^ An increase in the expression of *KATNA1*, *STMN1* and *KIF2C* genes indicates enhanced microtubule dynamics. *MAP2* and *MAPT* are both of these proteins bind to microtubules and stabilize them. STAT3 participates in cell proliferation and differentiation through the activation of downstream genes. As a kinase, PAK1 activates various substrates and works synergistically with ERK1/2 in regulating cytoskeletal reorganization. CAMK4 and PKA, as signalling molecules can influence various cellular processes through phosphorylation. ERK1/2 plays a central role in numerous cellular signalling pathways via affecting cell growth and division. Moreover, the expression of signal transduction proteins STAT3, PAK1, CAMK4, PKA and ERK1/2 was significantly down regulated. Collectively, lycorine exerts a multifaceted influence on the cell cycle conduction pathway, culminating in mitotic arrest in MCF‐7 cells.^33^, ^35^

## 5 CONCLUSIONS

In summary, this study uncovered a mechanism by lycorine induced BC through regulating cell‐cycle arrest. Activation of p21/CDK1/CyclinB1 signalling pathway involved in underlying mitotic cell cycle arrest in MCF‐7 cells was validated. Obviously, lycorine disrupted tubulin morphology and the dynamic balance of tubulin polymerization and depolymerization. The inhibitory activity of lycorine in MCF‐7 cells by which led to M‐phase cell cycle arrest, DNA damage and enhance microtubule dynamics was demonstrated for the first time.

Furthermore, these results imply that lycorine has the great prospect in the clinical treatment of BC and also has great significance for enriching the application of TCM in the development of new anti‐tumour drugs. Notably, TCM focuses on the characteristics of multi‐target and multi‐mechanism treatment of diseases, and thus the potential effect of lycorine on BC might be mediated by cell cycle arrest in M phase via targeting multiple tumorigenic pathways. Further studies will carry out the potential molecular mechanism of therapeutic action of lycorine on BC by microRNAs, long non‐coding RNAs and circular RNAs. Last but not least, it would be the consequence of exploitation of lycorine as a potential drug for BC therapy, however further preclinical and clinical studies are still needed.

## AUTHOR CONTRIBUTIONS

**Qinbing Xue:** Conceptualization (equal); methodology (equal); software (equal); writing – original draft (equal). **Bing Wang:** Data curation (equal); software (equal). **Jie Feng:** Conceptualization (equal); methodology (equal); software (equal); writing – original draft (equal). **Chaoyu Li:** Data curation (equal); software (equal). **Miao Yu:** Conceptualization (equal); funding acquisition (equal); supervision (equal). **Yan Zhao:** Data curation (equal); software (equal). **Zheng Qi:** Conceptualization (equal); supervision (equal); writing – review and editing (equal).

## FUNDING INFORMATION

This study was supported by the National Natural Science Foundation of China (Grant number: 41702368), the Key Research and Development Program of Heilongjiang Province (Grant number: 2022ZX02C07), the Young Innovative Talents Training Program of Harbin University of Commerce (Grant number: 2023‐KYYWF‐1032), and the Jinghu Scholar Support Program Project of Harbin University of Commerce (Grant number: 2023JHQNRC06).

## CONFLICT OF INTEREST STATEMENT

All authors declare no potential conflicts of interest.

## Supporting information


Data S1.

## Data Availability

The datasets generated and/or analyzed during the current study are available from the corresponding author on reasonable request.
