# Supplementary material for: Lycorine (Lycoris radiata)—a unique natural medicine on breast cancer
Source: J Cell Mol Med. 2024 Aug 22;28(16):e70032. doi: 10.1111/jcmm.70032 (PMC11341274; doi:10.1111/jcmm.70032)
Supplement: Supplementary file 1 — Data S1. [file JCMM-28-e70032-s001.docx]

Supplementary Materials

**Lycorine (*Lycoris radiata*)- A unique natural medicine on breast cancer**

Qinbing Xue^1^, Bing Wang^2^, Jie Feng^1^, Chaoyu Li^1^, Miao Yu^1,^*, Yan Zhao^3,^*, Zheng Qi^1,^*

^1^ Engineering Research Center for Medicine, Ministry of Education, Harbin University of Commerce, Harbin, China.

^2^ School of Food Engineering, Harbin University of Commerce, Harbin, China

^3^ Medical Imaging Department, The Fourth Affiliated Hospital of Harbin Medical University, Harbin, China

**Corresponding author:**

*Miao Yu and Zheng Qi

No. 138, Tongda Road, Daoli District, Harbin, 150076, China.

Tel.: +86-451-84866922; Fax: +86-451-84866922

E-mail: [yumiao913@163.com](mailto:yumiao913@163.com); [18645039597@163.com](mailto:18645039597@163.com); [qizheng@hrbcu.edu.cn](mailto:qizheng@hrbcu.edu.cn)

Qinbing Xue and Jie Feng contributed equally to this work.

**Tables**

**Table S1** Instruments and manufacturers

**Table S2** Reagents and manufacturers

**Table S3** Inhibition rate of lycorine on proliferation of MCF-7 cells

**Table S4** Primer sequences in PCR reaction system

**Table S5** Effects of lycorine on tumour weights of S180 mice

**Table S6** Effect of cycle arrest on protein expressions induced by lycorine

**Table S7** Classification and proportion of proteins in cell cycle arrest calculated by PCA analysis

**Table S8** Correlation analysis between mitosis protein and relative abundance of genes

**Figures**

**Fig. S1** The certification of the human MCF-7 cell line, human hepatoma cell line HepG2, human breast carcinoma cell line MDA-MB-231, human gastric carcinoma cell line SGC-7901 and non-small cell lung cancer cell line A549 contrast result detected by National Infrastructure of Cell Line Resource

**Fig. S2** 1D-NMR spectra of lycorine

**Fig. S3** Affidavit of approval of animal ethics and welfare

**Fig. S4** Primer sequences in PCR reaction system

**Fig.S5** Effect of MCF-7 cell cycle arrest on protein expressions induced by lycorine

**Table S1** Instruments and manufacturers

| Instruments | Manufacturer |
| --- | --- |
| CO-150 incubator | SANYO Electric Co., Ltd. |
| EPED-E2 - 30TJ Laboratory Super pure Water Apparatus | Nanjing Yipu Yida Technology Development Co., Ltd. |
| CKX-41 - 32 inverted microscope | Olympus Corporation, Japan |
| One thousandth of Adventurer electronic balance | Ohous, USA |
| DK-8D electric-heated thermostatic waterbath | Shanghai Yiheng Technology Instrument Co., Ltd. |
| Standard PB-10 pH Meter | Sartorius, Germany |
| DL-CJ-1N Medical Super Cleaning Worktable | Beijing HDL apparatus Co., Ltd. |
| 680 automatic microplate reader | Bio-Rad Laboratories, Inc. |
| Fluorescence microscope | Leica, Germany |
| Electrophoresis instrument DYY-7C | Beijing Liuyi Biotechnology Co., Ltd. |
| Vertical electrophoresis tank | Beijing Liuyi Biotechnology Co., Ltd. |
| TS-1000 Oscillator | Haimen Qilinbeier Instrument Manufacturing Co., Ltd. |
| Anke TDL80 - 2C Centrifuge | Shanghai Anting Scientific Instrument Factory |
| ImageQuant LAS500 Gel Imaging System | Shanghai Tanon Power Co., Ltd. |
| EPICS-XL Flowcytometer | Beckman Coulter, Inc., USA |
| Allegra 64R Tabletop High Speed Refrigerated Centrifuge | Beckman Coulter, Inc., USA |
| C-4040ZOOM Digital Camera | Olympus Corporation, Japan |
| Step One PlusTM Real-Time PCR system | Wcgene Biotech, Inc., Shanghai, China |

**Table S2** Reagents and manufacturers

| Reagents | Manufacturer |
| --- | --- |
| RPMI-1640 culture powder (batch number: ab37672)  Dulbecco’s modified Eagle’s medium (batch number: ab37654) | Gibco Life Technologies, Inc., USA  Gibco Life Technologies, Inc., USA |
| Lycorine (batch number: L413074) | Shanghai Aladdin Biochemical Technology Co., Ltd. |
| Fetal bovine serum (batch number: 20160402a) | Corning Life Co., Ltd. New Zealand |
| Trypsin (batch number: 20150703) | Gibco Life Technologies, Inc., USA |
| DMSO (batch number: 20160204) | Tianjin Zhonghe Shengtai Chemical Co., Ltd. |
| MTT (batch number: 20151121) | Jiangsu Beyotime Institute of Biotechnology |
| Penicillin-Streptomycin Solution (100X) (batch number: C0222） | Jiangsu Beyotime Institute of Biotechnology |
| NaCl (batch number: bs130727) | Shanghai Chemical Reagent Company |
| KCl (batch number: bs130402) | Tianjin Corrine Chemical Sales Company |
| KH_2_PO_4_ (batch number: 20130301) | Weifang Hengfeng Chemical Co., Ltd. |
| Na_2_HPO_4_ (batch number: 20130517) | Shanghai Chemical Reagent Company |
| Propidium iodide (PI) (batch number: 20160102) | Sigma-Aldrich LLC., USA |
| Ethanol (95%) (batch number: 20160309) | Shandong Yinzhou Chemical Co., Ltd. |
| Triton-X-100 (batch number: 114-a) | Farco, Hung |
| Sodium Citrate (batch number: 20120317) | Beijing Chemical Reagent Company |
| Ribonuclease A (batch number: 20160304) | Beijing Chemical Reagent Company |
| NaHCO_3_ (batch number: 20140508) | Tianjin BASF Chemical Trading Co., Ltd. |
| Saline (batch number: 20160414) | Harbin Triple Pharmaceutical Co., Ltd. |
| Crystal violet staining solution (batch number: C0121) | Shanghai Beyotime Biological Co.,Ltd. |
| Glycerol (batch number: 20130703)  Cyclophosphamide (batch number: S83332)  Vincristine (VCR) (batch number: B20160) | Shanghai Macklin Biochemical Technology Co., Ltd.  Shanghai Yuanye Biotechnology Co.,Ltd.  Shanghai Yuanye Biotechnology Co.,Ltd. |
| TriTris (batch number: 0479) | Sigma-Aldrich LLC., USA |
| Glycine (batch number: ST085) | Jiangsu Beyotime Institute of Biotechnology |
| BCA protein concentration determination kit (batch number: P0010S) | Jiangsu Beyotime Institute of Biotechnology |
| DAB Substrate Kit (batch number: P0203) | Beijing ZhongShan-Golden Bridge Biological Technology |
| Skimmed milk powder (batch number: CM0004) | Hefei Lanxu Biotechnology Co., Ltd. |
| Acrylamide (batch number: A012901) | Beijing Huamaike Biotechnology Co., Ltd. |
| IgG/HRP (batch number : ZDR-5306） | Jiangsu Beyotime Institute of Biotechnology |
| Rabbit Anti-β actin mAb (batch number : TA-09） | Jiangsu Beyotime Institute of Biotechnology |
| Rabbit Anti-Cyclin B1 antibody (batch number : bs-0572R） | Jiangsu Beyotime Institute of Biotechnology |
| Rabbit Anti-CDK1 antibody (batch number : bs-1341R） | Jiangsu Beyotime Institute of Biotechnology |
| Rabbit Anti-p21 antibody (batch number : bs-2687R） | Biosynthesis Biotechnology Co., Ltd. |
| Rabbit Anti-Aurora A antibody (batch number: bsm-52018R）  Rabbit Anti-Aurora B antibody (batch number : bs-22286R）  Rabbit Anti-p-H3 antibody (batch number : bs-3189R）  Rabbit Anti-PLK1 antibody (batch number : bsm-33865M）  Rabbit Anti-BRCA1 antibody (batch number : bs-20490R）  Rabbit Anti-STAT3 antibody (batch number : bsm-52235R）  Rabbit Anti-PAK1 antibody (batch number : bs-2277R）  Rabbit Anti-CAMK4 antibody (batch number : bs-6727R）  Rabbit Anti-PKA antibody (batch number : bs-3964R）  Rabbit Anti-ERK1/2 antibody (batch number : bsm-52259R） | Beijing Bioss Biotechnology Co., Ltd.  Beijing Bioss Biotechnology Co., Ltd  Beijing Bioss Biotechnology Co., Ltd  Beijing Bioss Biotechnology Co., Ltd  Beijing Bioss Biotechnology Co., Ltd  Beijing Bioss Biotechnology Co., Ltd  Beijing Bioss Biotechnology Co., Ltd  Beijing Bioss Biotechnology Co., Ltd  Beijing Bioss Biotechnology Co., Ltd  Beijing Bioss Biotechnology Co., Ltd |
| Rabbit Anti-BubR1 antibody (batch number : bs-5726R） | Biosynthesis Biotechnology Co., Ltd. |
| Color-coded Prestained Protein Marker (10-180 kDa (batch number: P0068） | Jiangsu Beyotime Institute of Biotechnology |
| Ponceau Staining Solution (batch number: P0022） | Jiangsu Beyotime Institute of Biotechnology |
| Ammonium persulfate (APS) (batch number: ST005) | Jiangsu Beyotime Institute of Biotechnology |
| Tween-20 (batch number: ST825) | Jiangsu Beyotime Institute of Biotechnology |
| PMSF (100mM) (batch number: ST506) | Jiangsu Beyotime Institute of Biotechnology |
| TEMED (batch number: ST728) | Jiangsu Beyotime Institute of Biotechnology |
| SDS (batch number: BOO0276) | Sigma-Aldrich LLC., USA |
| DTT (batch number: ST040) | Jiangsu Beyotime Institute of Biotechnology |
| Bromophenol Blue (batch number: B802654) | Shanghai Macklin Biochemical Technology Co., Ltd |
| Methanol (batch number: 20160509) | Guangzhou Yuebao Chemical Technology Co., Ltd. |

**Table S3** Inhibition rate of lycorine on proliferation of MCF-7 cells (*n*=6)

| Groups | Concentration (μmol/L) | Optical density | Inhibitory rate (%) |
| --- | --- | --- | --- |
| Control | - | 1.355±0.07 | - |
| Lycorine | 1 | 1.187±0.13** | 14.9 |
|  | 2 | 1.075±0.09** | 24.8 |
|  | 4 | 0.974±0.16** | 33.7 |
|  | 8 | 0.929±0.05** | 37.7 |
|  | 16 | 0.777±0.04** | 51.2 |
|  | 32 | 0.632±0.03** | 64.1 |
| VCR | 0.625 | 0.892±0.05** | 52.7 |

Significant difference compared with control group, ^**^*P* <0.01

**Table S4** Primer sequences in PCR reaction system

| **ARGs** | Forward primer | Reverse primer |
| --- | --- | --- |
| ***ABL1*** | TGGTCCCTTCCTTTTGTTAACGT | TATGCACACGCCACTTAGAAAAGA |
| ***ANAPC2*** | TATGTTGCGCGGAGTCTTGTT | GAAGCACCCATACAGACGCTG |
| ***ATM*** | ATCTGCTGCCGTCAACTAGAA | GATCTCGAATCAGGCGCTTAAA |
| ***ATR*** | ACATTTGTGACTGGAGTAGAAGA | TCCACAATTGGTGACCTGGG |
| ***AURKA*** | TGGGTGGTCAGTACATGCTC | TGCATCCGACCTTCAATCATTTC |
| ***AURKB*** | CGCAGAGAGATCGAAATCCAG | AGATCCTCCTCCGGTCATAAAA |
| ***BCCIP*** | ACAGAACCAATAAGCCATGTGG | TGCAAACATTAACGCAGCTTTC |
| ***BCL2*** | ATTGATGGGATCGTTGCCTTAT | TCCAATTCCTTTCGGATCTTTA |
| ***BIRC5*** | TGGGAAGGGTTGTGAATGAG | CAGTTTGGCTTGCTGGTCTC |
| ***BRCA1*** | GAAACCGTGCCAAAAGACTTC | CCAAGGTTAGAGAGTTGGACAC |
| ***BRCA2*** | TGCCTGAAAACCAGATGACTATC | AGGCCAGCAAACTTCCGTTTA |
| ***CASP3*** | TGGTTCATCCAGTCGCTTTGT | CCCGGGTAAGAATGTGCATAAA |
| ***CCNA2*** | TGGAAAGCAAACAGTAAACAGCC | GGGCATCTTCACGCTCTATTT |
| ***CCNB1*** | AATAAGGCGAAGATCAACATGGC | TTTGTTACCAATGTCCCCAAGAG |
| ***STMN1*** | TCAGCCCTCGGTCAAAAGAAT | TTCTCGTGCTCTCGTTTCTCA |
| ***KIF2C*** | GAGAGCAAGCTGACACAGGT | CCTGGTGAGATCGTGGCAAT |
| ***KATNA1*** | AGCACTCCCTTGAAAGCGG | GCGTTTTCTAGGTCCTGGTGA |
| ***CCNB2*** | TGCTCTGCAAAATCGAGGACA | GCCAATCCACTAGGATGGCA |
| ***CCNC*** | CCTTGCATGGAGGATAGTGAATG | AAGGAGGATACAGTAGGCAAAGA |
| ***CCND1*** | GCTGCGAAGTGGAAACCATC | CCTCCTTCTGCACACATTTGAA |
| ***CCND2*** | CTGTCTCTGATCCGCAAGCAT | GGTGGGTACATGGCAAACTTAAA |
| ***CCND3*** | TACCCGCCATCCATGATCG | AGGCAGTCCACTTCAGTGC |
| ***CCNE1*** | AAGGAGCGGGACACCATGA | ACGGTCACGTTTGCCTTCC |
| ***CCNF*** | CACAAAGCATCCATATTGCACTG | TGGTCAGACATCCCTGATGAG |
| ***CCNG1*** | GAGTCTGCACACGATAATGGC | GTGCTTGGGCTGTACCTTCA |
| ***CCNG2*** | TCTCGGGTTGTTGAACGTCTA | GTAGCCTCAATCAAACTCAGCC |
| ***CCNH*** | AGGCACTTGAACAGATACTGGA | CCAATATGGGATAGCGGGTCT |
| ***CCNT1*** | ACAACAAACGGTGGTATTTCACT | CCTGCTGGCGATAAGAAAGTT |
| ***CDC16*** | CTTGAAGGACGAAAGTGGCTT | TTCCCGCGTAGAAGACAGATA |
| ***CDC20*** | GCTTTGAACCTGAACGGTTTTG | TCTGGCGCATTTTGTGGTTTT |
| ***CDC25A*** | GTGAAGGCGCTATTTGGCG | TGGTTGCTCATAATCACTGCC |
| ***STAT3*** | CAGCAGCTTGACACACGGTA | AAACACCAAAGTGGCATGTGA |
| ***ERK*** | TCACACAGGGTTCCTGACAGA | ATGCAGCCTACAGACCAAATATC |
| ***PAK1*** | AGGGGAGTTTACGGGAATGC | TCTTCTGCTCCGACTTAGTGATA |
| ***CDC25C*** | TCTACGGAACTCTTCTCATCCAC | TCCAGGAGCAGGTTTAACATTTT |
| ***CDC34*** | AACGAGCCCAACACCTTCTC | GTACTCCCGATCCTTCCCCT |
| ***CDC6*** | TGTTCTCCTCGTGTAAAAGCC | GGGGAGTGTTGCATAGGTTGT |
| ***CDK1*** | GGATGTGCTTATGCAGGATTCC | CATGTACTGACCAGGAGGGATAG |
| ***CDK2*** | GTACCTCCCCTGGATGAAGAT | CGAAATCCGCTTGTTAGGGTC |
| ***CDK4*** | TCAGCACAGTTCGTGAGGTG | GTCCATCAGCCGGACAACAT |
| ***CDK5R1*** | AGAACAGCAAGAACGCCAAG | CGGCCACGATTCTCTTCCA |
| ***CDK5RAP1*** | ATGGCTGCCAGATGAATGTGA | CTCTTGGAGGTTACTGGTCCG |
| ***CDK6*** | CCAGATGGCTCTAACCTCAGT | AACTTCCACGAAAAAGAGGCTT |
| ***CDK7*** | GGAGCCCCAATAGAGCTTATACA | TCCACACCTACACCATACATCC |
| ***CDK8*** | GGGATCTCTATGTCGGCATGT | CACACCTTCCTATCAGCATGAG |
| ***CDKN1A*** | TGTCCGTCAGAACCCATGC | AAAGTCGAAGTTCCATCGCTC |
| ***CDKN1B*** | ATCACAAACCCCTAGAGGGCA | GGGTCTGTAGTAGAACTCGGG |
| ***CDKN2A*** | ATGGAGCCTTCGGCTGAC | GTAACTATTCGGTGCGTTGG |
| ***CAMK4*** | GCCTCGTCCCGGATTACTG | TCCCCTTCTGTTTGCATCTGT |
| ***CDKN2B*** | GGGACTAGTGGAGAAGGTGC | CATCATCATGACCTGGATCGC |
| ***CDKN3*** | AGCCGCCCAGTTCAATACAA | CCTGGAAGAGCACATAAACCG |
| ***CHEK1*** | ATATGAAGCGTGCCGTAGACT | TGCCTATGTCTGGCTCTATTCTG |
| ***CHEK2*** | TGAGAACCTTATGTGGAACCCC | ACAGCACGGTTATACCCAGC |
| ***CKS1B*** | TATTCGGACAAATACGACGACG | CGCCAAGATTCCTCCATTCAGA |
| ***CKS2*** | TTCGACGAACACTACGAGTACC | GGACACCAAGTCTCCTCCAC |
| ***CUL1*** | AGCCATTGAAAAGTGTGGAGAA | GCGTCATTGTTGAATGCAGACA |
| ***CUL2*** | ACGACAATAAAAGCCGTGGTC | GGATAGGCCACACATAAAGCAT |
| ***CUL3*** | TGTGGAGAACGTCTACAATTTGG | GCGCCTCTGTCTACGACTT |
| ***E2F1*** | CATCCCAGGAGGTCACTTCTG | GACAACAGCGGTTCTTGCTC |
| ***E2F4*** | CCTAGCTGTACGCCAGAAGC | TTTTTCTCGATTAGCCCGATACC |
| ***GADD45A*** | GGATGCCCTGGAGGAAGTG | CTTCGTACACCCCGACAGTGA |
| ***GTSE1*** | CAGGGGACGTGAACATGGATG | ATGTCCAAAGGGTCCGAAGAA |
| ***HUS1*** | GAATGCCAGGGCTTTGAAAATC | CACAATGCGGCTACTGCTTG |
| ***TPPP3*** | TCCATTCCTGCGTCGTTCTG | TTCATCTCTTGCCCACTGGC |
| ***MAP2*** | CTCAGCACCGCTAACAGAGG | CATTGGCGCTTCGGACAAG |
| ***MAPT*** | CCAAGTGTGGCTCATTAGGCA | CCAATCTTCGACTGGACTCTGT |
| ***KNTC1*** | GAGAGAAGTGGCAACCTACATC | CGCCGATTTTCATCGTTAGCTTT |
| ***KPNA2*** | GGCACTGTAAATTGGTCTGTTGA | CCTGGCAGCTTGAGTAGCTT |
| ***MAD2L1*** | GTTCTTCTCATTCGGCATCAACA | GAGTCCGTATTTCTGCACTCG |
| ***MAD2L2*** | ACCGCCCAGTGGAGAAATTC | CATCGCACACGCTGATCTT |
| ***MCM2*** | ATGGCGGAATCATCGGAATCC | GGTGAGGGCATCAGTACGC |
| ***MCM3*** | TCAGAGAGATTACCTGGACTTCC | TCAGCCGGTATTGGTTGTCAC |
| ***MCM4*** | CACCACACACAGTTATCCTGTT | CGAATAGGCACAGCTCGATAGAT |
| ***MCM5*** | AGCATTCGTAGCCTGAAGTCG | CGGCACTGGATAGAGATGCG |
| ***MDM2*** | CAGTAGCAGTGAATCTACAGGGA | CTGATCCAACCAATCACCTGAAT |
| ***MKI67*** | CGTCCCAGTGGAAGAGTTGT | CGACCCCGCTCCTTTTGATA |
| ***MNAT1*** | AGCTCTGTATGAATACCAGCCA | AGTCTTCCTAGCATCTCAAGCTC |
| ***MRE11A*** | ATCGGCCTGTCCAGTTTGAAA | TGCCATCTTGATAGTTCACCCAT |
| ***NBN*** | GACTGGCGTTGAGTACGTTGT | TGATTTCGGCTGATCGACTGA |
| ***RAD1*** | CTCAACAGTGGGGTAGGCAG | GGTGGGGATAAGGGACAAGC |
| ***DIAPH1*** | CTTGCGGGATATGCCTCTG | AATGTTTGCACCCAACTGACA |
| ***RAC1*** | GTGCAGACACTTGCTCTCCT | AATGGCAACGCTTCATTCGG |
| ***CDC42*** | CCATCGGAATATGTACCGACTG | CTCAGCGGTCGTAATCTGTCA |
| ***RAD17*** | ACACGCTCTTTACTCAGGGAA | AGCATATCCTCGGGCTTTGTT |
| ***RAD51*** | CAACCCATTTCACGGTTAGAGC | TTCTTTGGCGCATAGGCAACA |
| ***RAD9A*** | CATTGACTCTTACATGATCGCCA | GCCAGGTGAAAGGGAAATGG |
| ***RB1*** | TTGGATCACAGCGATACAAACT | AGCGCACGCCAATAAAGACA |
| ***RBBP8*** | AGATCGGTTAAGAGCAGGCTT | TGCTGCCGGATATTTTCAAACT |
| ***RBL1*** | TCGGATGATTGGGGATGACTT | GCACATAATCGCATTGGCAAAAA |
| ***RBL2*** | TGAGCGAAAGCTACACGCTG | TCCCTTTGCTTACAGTTGGAAC |
| ***SERTAD1*** | CTGGCTGTCTACTGGACGATG | TGGTGCCCAAAGTTCATTGTC |
| ***SKP2*** | ATGCCCCAATCTTGTCCATCT | CACCGACTGAGTGATAGGTGT |
| ***TFDP1*** | AGGGCCTACGGCATTTCTC | CTCGTCTGCCACTTCGTTGT |
| ***TFDP2*** | CTGCCTACCAATTCTGCTCAG | CGCTTCTGCTTTATCCGTTCT |
| ***TP53*** | GAGGTTGGCTCTGACTGTACC | TCCGTCCCAGTAGATTACCAC |
| ***WEE1*** | TGAAGAGGCTGGATGGATGC | TTCTGCCCACGCAGAGAAAT |
| ***RHOA*** | GGAAAGCAGGTAGAGTTGGCT | GGCTGTCGATGGAAAAACACAT |
| ***CKAP5-A*** | TGTGGAAAGCAAGGTTAAGTGG | ACTCTGGGCTCTTTTCATCCT |
| ***PKA*** | AGCCCACTTGGATCAGTTTGA | GTTCCCGGTCTCCTTGTGT |
| ***ICIS*** | CCGGGGAGAGCTAGTCACT | CTGCTGGACGAATGCTTCA |
| ***CDC2*** | GGATGTGCTTATGCAGGATTCC | CATGTACTGACCAGGAGGGATAG |

**Table S5** Effects of lycorine on tumor weights of S180 mice (*n*=10)

| Group | Dose (mg/kg) | Tumor weigh (g) | Inhibition Rate (%) |
| --- | --- | --- | --- |
| Control | — | 1.15±0.11 | — |
| CTX | 10 | 0.49±0.08^**^ | 56.84 |
| Lycorine | 10 | 0.89±0.12^**^ | 21.69 |
| Lycorine | 20 | 0.78±0.17^**^ | 32.07 |
| Lycorine | 40 | 0.57±0.13^**^ | 50.58 |

Significant difference compared with control group, ^**^*P* <0.01

**Table S6** Effect of cycle arrest on protein expressions induced by lycorine (n=3).

| **Proteins** | Control | VCR | 7 μmol/L | 14 μmol/L | 28 μmol/L |
| --- | --- | --- | --- | --- | --- |
| CyclinB1/β-Actin | 1.08±0.01 | 0.65±0.00^***^ | 0.41±0.01^***^ | 0.38±0.01^***^ | 0.19±0.01^***^ |
| CDK1/β-Actin | 1.09±0.04 | 0.87±0.04^***^ | 1.02±0.01 | 0.75±0.01^***^ | 0.62±0.02^***^ |
| p21/β-Actin | 0.52±0.02 | 1.23±0.02^***^ | 0.45±0.01^***^ | 0.72±0.03^***^ | 0.75±0.02^***^ |
| Aurora A/β-Actin | 0.91±0.03 | 0.29±0.01^***^ | 0.92±0.01 | 0.79±0.01^**^ | 0.77±0.00^***^ |
| Aurora B/β-Actin | 1.22±0.01 | 0.55±0.01^***^ | 0.76±0.01^***^ | 0.69±0.00^***^ | 0.62±0.01^***^ |
| STAT3/GADPH | 1.65±0.05 | 1.25±0.06^***^ | 1.45±0.05 | 0.93±0.07^***^ | 0.82±0.03^***^ |
| PAK1/GADPH | 1.45±0.05 | 1.18±0.03^**^ | 0.92±0.07^***^ | 0.81±0.03^***^ | 0.75±0.02^***^ |
| CAMK4/GADPH | 1.3±0.02 | 0.92±0.03^*^ | 1.13±0.05 | 0.9±0.08^*^ | 0.65±0.03^**^ |
| PKA/GADPH | 1.15±0.02 | 0.52±0.02^***^ | 0.85±0.03^***^ | 0.75±0.02^***^ | 0.5±0.04^***^ |
| ERK1/GADPH | 0.57±0.05 | 0.37±0.02^*^ | 0.41±0.03 | 0.38±0.02^*^ | 0.34±0.04^**^ |
| ERK2/GADPH | 0.62±0.02 | 0.36±0.02^*^ | 0.40±0.05 | 0.37±0.03^*^ | 0.33±0.02^*^ |

In each cell, the value indicates the *P*-value, ^*^ *P*＜0.05, ^**^ *P*＜0.01, ^***^ *P*＜0.001.

**Table S7** Classification and proportion of proteins in cell cycle arrest calculated by PCA analysis

| **Proteins** | **PC1 (88.8%)** | **PC2 (6.4%)** | **PC3 (3.1%)** |
| --- | --- | --- | --- |
| CyclinB1 | -1.78564 | -0.04188 | **0.01567** |
| CDK1 | -3.8643 | -0.69761 | **-0.01461** |
| p21 | -3.03271 | **1.41814** | -0.48869 |
| Aurora A | -1.14403 | -0.41752 | **0.09986** |
| Aurora B | -2.40351 | -0.99334 | **0.1933** |
| p-H3 | -5.08589 | -0.28909 | **0.34799** |
| PLK1 | **0.38954** | -0.79803 | -0.21024 |
| BRCA1 | 0.16248 | 0.52092 | **1.51571** |
| BUBR1 | 0.11785 | **2.83211** | -0.39083 |
| STAT3 | **7.72889** | -0.34553 | 0.0857 |
| PAK1 | **5.42342** | 0.19241 | 1.08569 |
| CAMK4 | **5.00192** | -0.24517 | -0.58637 |
| PKA | **2.22545** | -0.53335 | -1.46269 |
| EKR1 | -1.84439 | -0.21735 | **-0.05468** |
| ERK2 | -1.88909 | -0.38472 | **-0.13583** |

**Table S8** Correlation analysis between mitosis protein and relative abundance of genes

|  | CyclinB1 | CDK1 | p21 | Aurora A | Aurora B | p-H3 | PLK1 |
| --- | --- | --- | --- | --- | --- | --- | --- |
| *CCNB2* | 0.270 | 0.051 | 0.064 | 0.484 | 0.205 | 0.495 | 0.936 |
| *CCNF* | 0.076 | **0.010*** | **0.014*** | 0.185 | 0.057 | 0.239 | 0.688 |
| *CDC25C* | 0.083 | **0.011*** | **0.016*** | 0.194 | 0.063 | 0.244 | 0.684 |
| *CDC6* | 0.234 | **0.032*** | **0.044*** | 0.473 | 0.172 | 0.497 | 0.863 |
| *CDC20* | 0.310 | **0.049*** | **0.064*** | 0.574 | 0.231 | 0.578 | 0.780 |
| *MRE11A* | 0.590 | 0.153 | 0.180 | 0.873 | 0.467 | 0.827 | 0.595 |
| *RAD51* | 0.102 | **0.017*** | **0.023*** | 0.212 | 0.078 | 0.251 | 0.655 |
| *KIF2C* | **0.001**** | **0***** | **0***** | **0.001**** | **0.001**** | **0.002*** | **0.003*** |
| *STMN1* | **0***** | **0***** | **0***** | **0.001**** | **0.001**** | **0.002*** | **0.003*** |
| *KATNA1* | **0***** | **0***** | **0***** | **0***** | **0***** | **0.001**** | **0.001**** |
| *MAP2* | 0.261 | **0.037*** | 0.050 | 0.512 | 0.192 | 0.528 | 0.825 |
| *MAPT* | 0.124 | **0.018*** | **0.024*** | 0.270 | 0.093 | 0.314 | 0.820 |
| *PAK1* | **0.034*** | **0.004**** | **0.006**** | 0.096 | **0.027*** | 0.150 | 0.490 |
| *Stat3* | **0.001**** | **0***** | **0***** | **0.007** | **0.001**** | **0.030*** | 0.117 |
| *ERK* | **0***** | **0***** | **0***** | **0.001**** | **0***** | **0.001**** | **0.001**** |
| *PKA* | **0.035*** | **0.004**** | **0.006**** | 0.103 | **0.028*** | 0.162 | 0.531 |
| *CAMK4* | **0.010*** | **0.001**** | **0.002**** | **0.034*** | **0.008**** | 0.074 | 0.271 |
| *ICIS* | 0.049 | **0.006**** | **0.008**** | 0.133 | **0.038*** | 0.190 | 0.591 |
| *AURKB* | **0.009**** | **0.001**** | **0.002**** | **0.033*** | **0.008**** | 0.076 | 0.282 |
| *CDC2* | **0.007**** | **0.001**** | **0.001**** | **0.029*** | **0.007**** | 0.072 | 0.273 |

In each cell, the value indicates the *P*-value. Bold values indicate statistical significance (*P*＜0.05), ^*^ *P*＜0.05, ^**^ *P*＜0.01, ^***^ *P*＜0.001.

**Table S8** Correlation analysis between mitosis protein and relative abundance of genes (continued table)

|  | BRCA1 | BUBR1 | STAT3 | PAK1 | CAMK4 | PKA | EKR1 | ERK2 |
| --- | --- | --- | --- | --- | --- | --- | --- | --- |
| *CCNB2* | 0.892 | 0.656 | **0.018*** | 0.056 | 0.063 | 0.403 | 0.276 | 0.291 |
| *CCNF* | 0.496 | 0.314 | **0.021*** | 0.075 | 0.086 | 0.602 | 0.081 | 0.092 |
| *CDC25C* | 0.499 | 0.320 | **0.023*** | 0.082 | 0.094 | 0.621 | 0.089 | 0.100 |
| *CDC6* | 0.946 | 0.674 | **0.012*** | **0.037*** | **0.040*** | 0.328 | 0.243 | 0.262 |
| *CDC20* | 0.954 | 0.776 | **0.011*** | **0.035*** | **0.038*** | 0.297 | 0.318 | 0.337 |
| *MRE11A* | 0.723 | 0.953 | **0.012*** | **0.034*** | **0.038*** | 0.240 | 0.592 | 0.607 |
| *RAD51* | 0.491 | 0.328 | **0.032*** | 0.110 | 0.129 | 0.692 | 0.107 | 0.117 |
| *KIF2C* | **0.002**** | **0.002**** | 0.276 | 0.072 | **0.047*** | **0.011*** | **0.001**** | **0.001**** |
| *STMN1* | **0.002**** | **0.001**** | 0.340 | 0.087 | 0.056 | **0.013*** | **0.001**** | **0.001**** |
| *KATNA1* | **0.001**** | **0.001**** | 0.095 | **0.023*** | **0.015*** | **0.004**** | **0***** | **0***** |
| *MAP2* | 0.989 | 0.715 | **0.011*** | **0.035*** | **0.038*** | 0.312 | 0.269 | 0.288 |
| *MAPT* | 0.629 | 0.420 | **0.020*** | 0.067 | 0.076 | 0.525 | 0.130 | 0.143 |
| *PAK1* | 0.317 | 0.187 | **0.026*** | 0.095 | 0.110 | 0.755 | **0.038*** | **0.045*** |
| *Stat3* | **0.046*** | **0.024*** | **0.039*** | 0.171 | 0.206 | 0.761 | **0.002**** | **0.003**** |
| *ERK* | **0.002**** | **0.001**** | 0.252 | 0.063 | **0.041*** | **0.010*** | **0.001**** | **0.001**** |
| *PKA* | 0.346 | 0.203 | **0.022*** | 0.083 | 0.094 | 0.701 | **0.039*** | **0.047*** |
| *CAMK4* | 0.147 | 0.081 | **0.034*** | 0.134 | 0.159 | 0.995 | **0.011*** | **0.015*** |
| *ICIS* | 0.403 | 0.244 | **0.022*** | 0.081 | 0.092 | 0.664 | 0.054 | 0.063 |
| *AURKB* | 0.152 | 0.082 | **0.031*** | 0.123 | 0.144 | 0.963 | **0.011*** | **0.014*** |
| *CDC2* | 0.141 | 0.076 | **0.029*** | 0.116 | 0.135 | 0.956 | **0.009**** | **0.012*** |

In each cell, the value indicates the *P*-value. Bold values indicate statistical significance *(P*＜0.05), ^*^ *P*＜0.05, ^**^ *P*＜0.01, ^***^ *P*＜0.001.


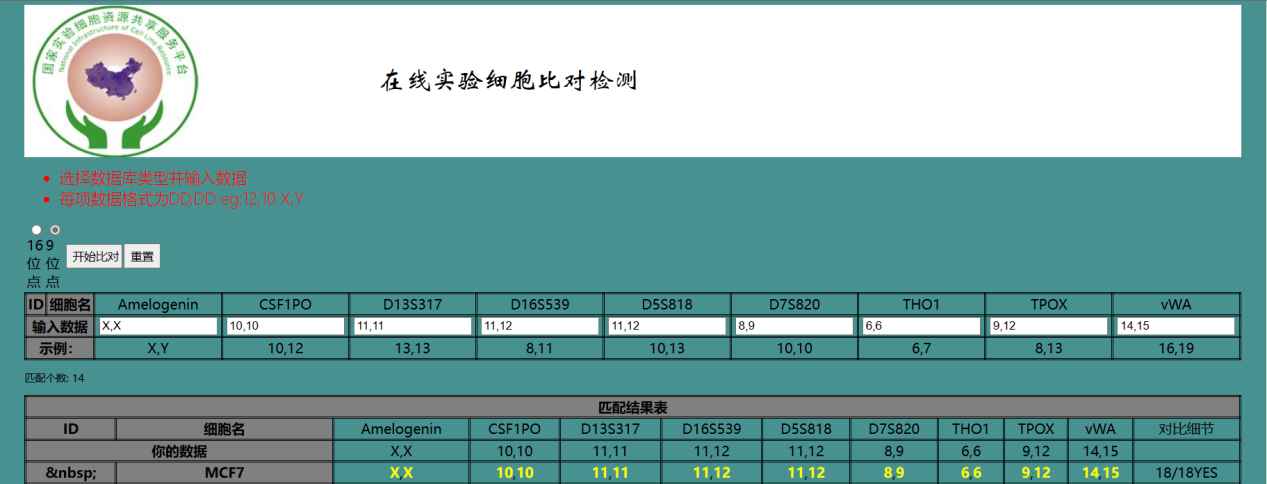


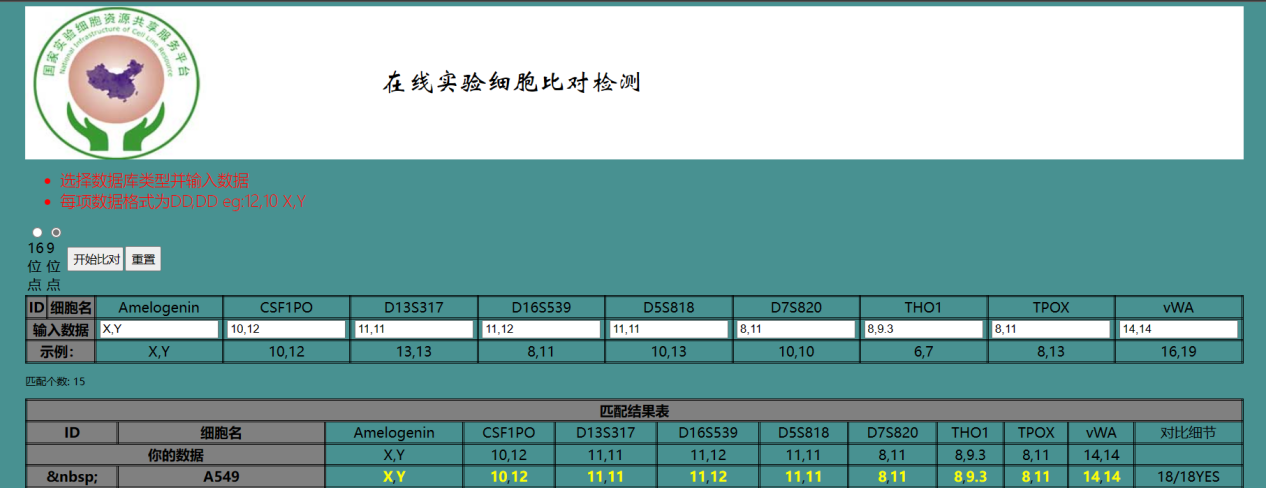


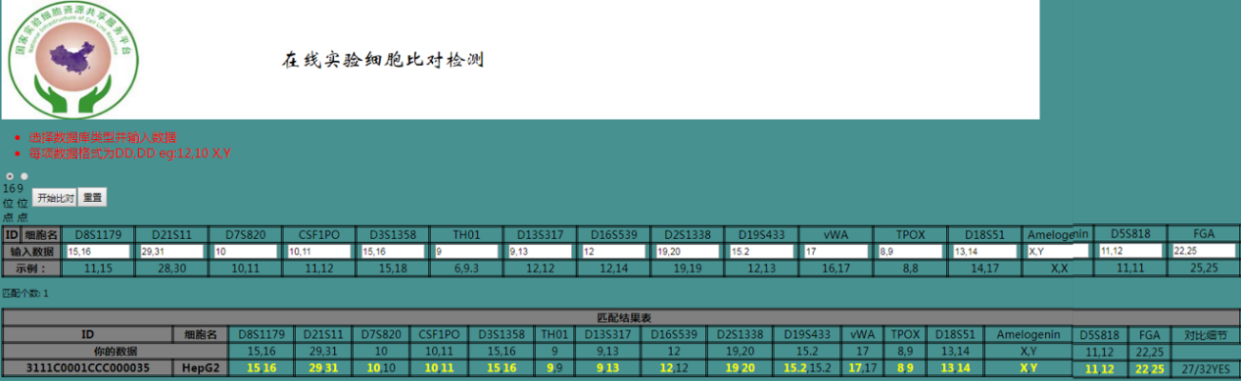


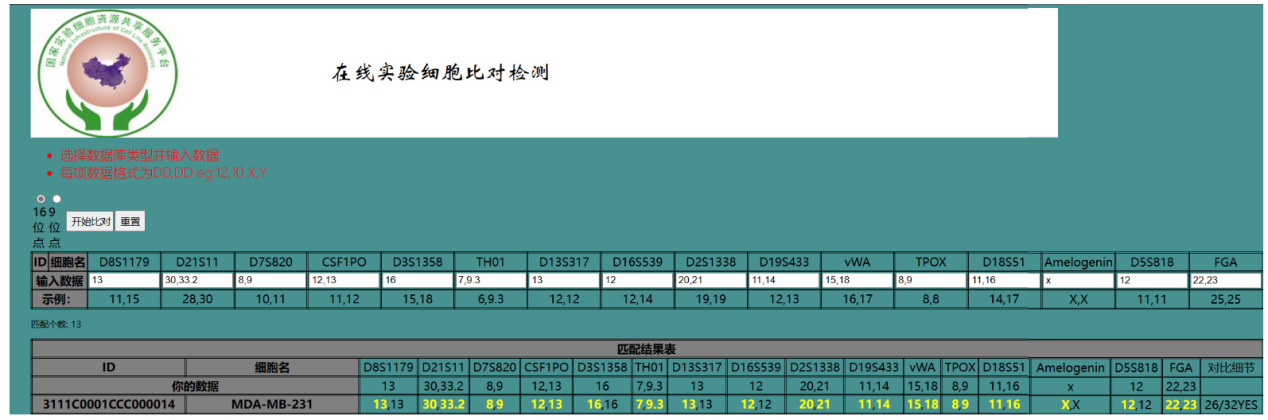


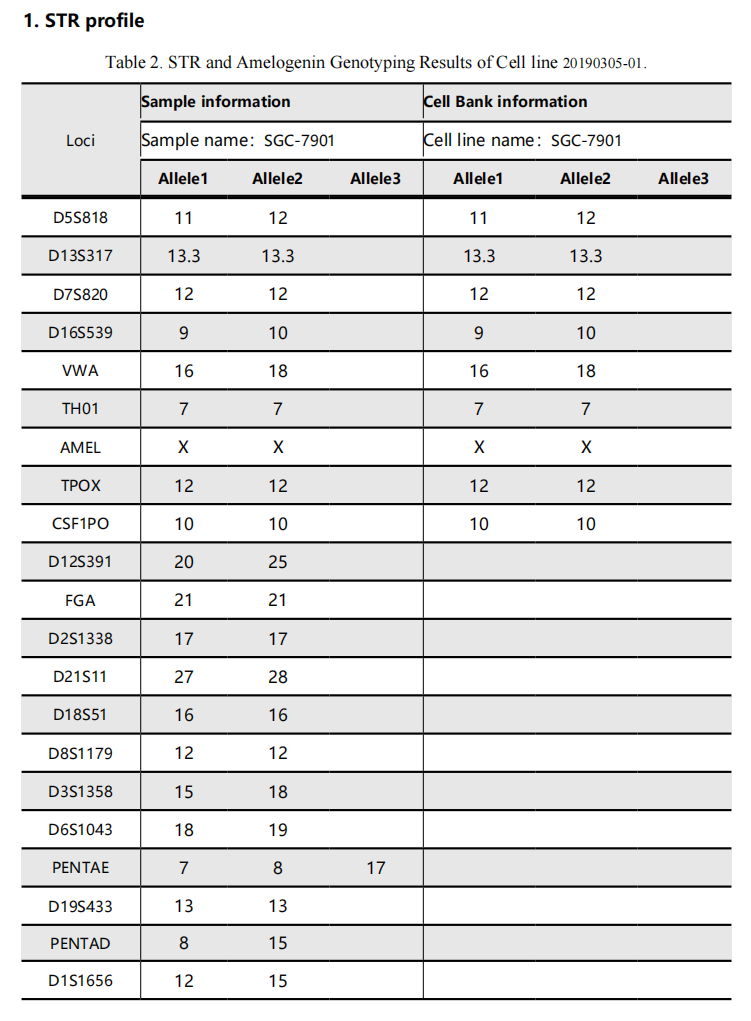


**Fig. S1** The certification of the human MCF-7 cell line, human hepatoma cell line HepG2, human breast carcinoma cell line MDA-MB-231, human gastric carcinoma cell line SGC-7901 and non-small cell lung cancer cell line A549 contrast result detected by National Infrastructure of Cell Line Resource


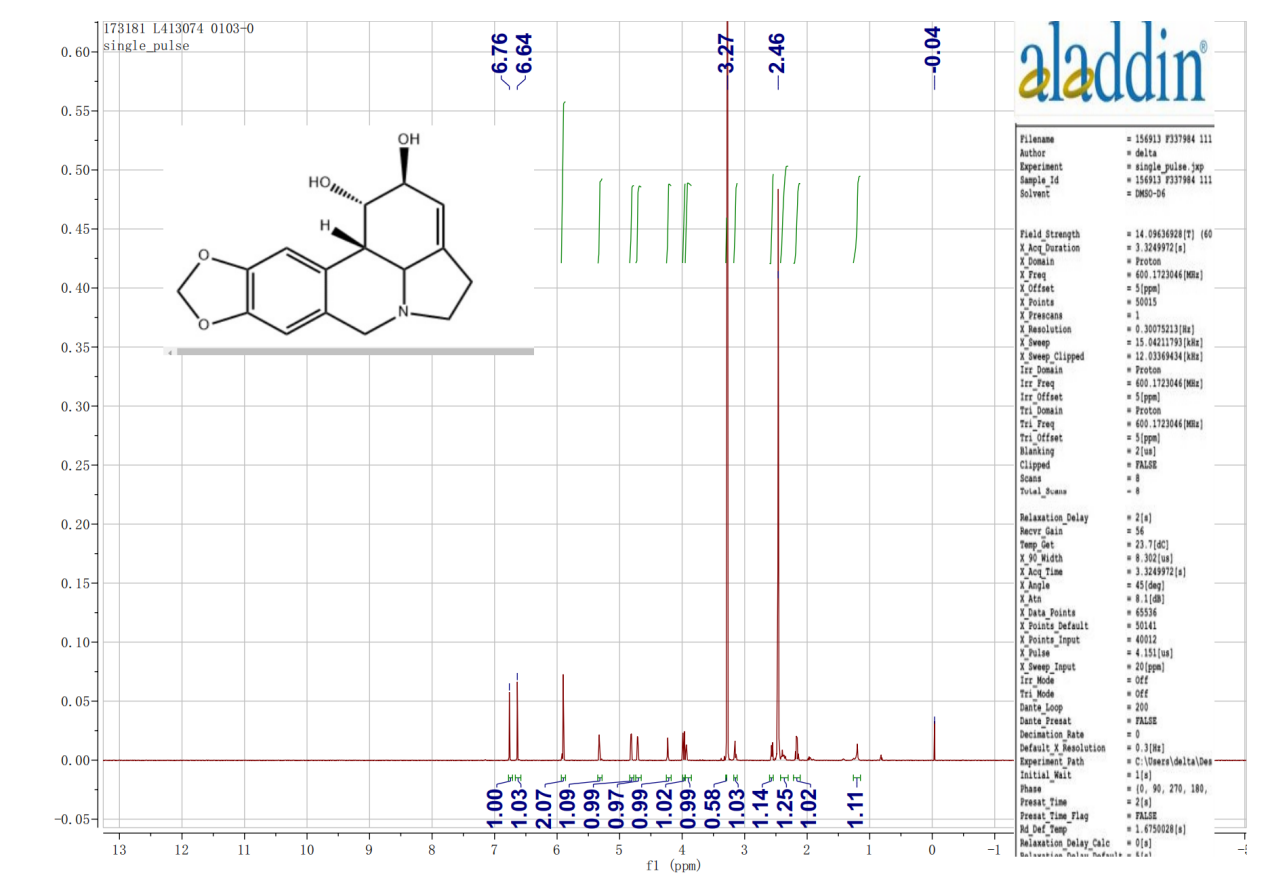


**Fig. S2** 1D-NMR spectra of lycorine


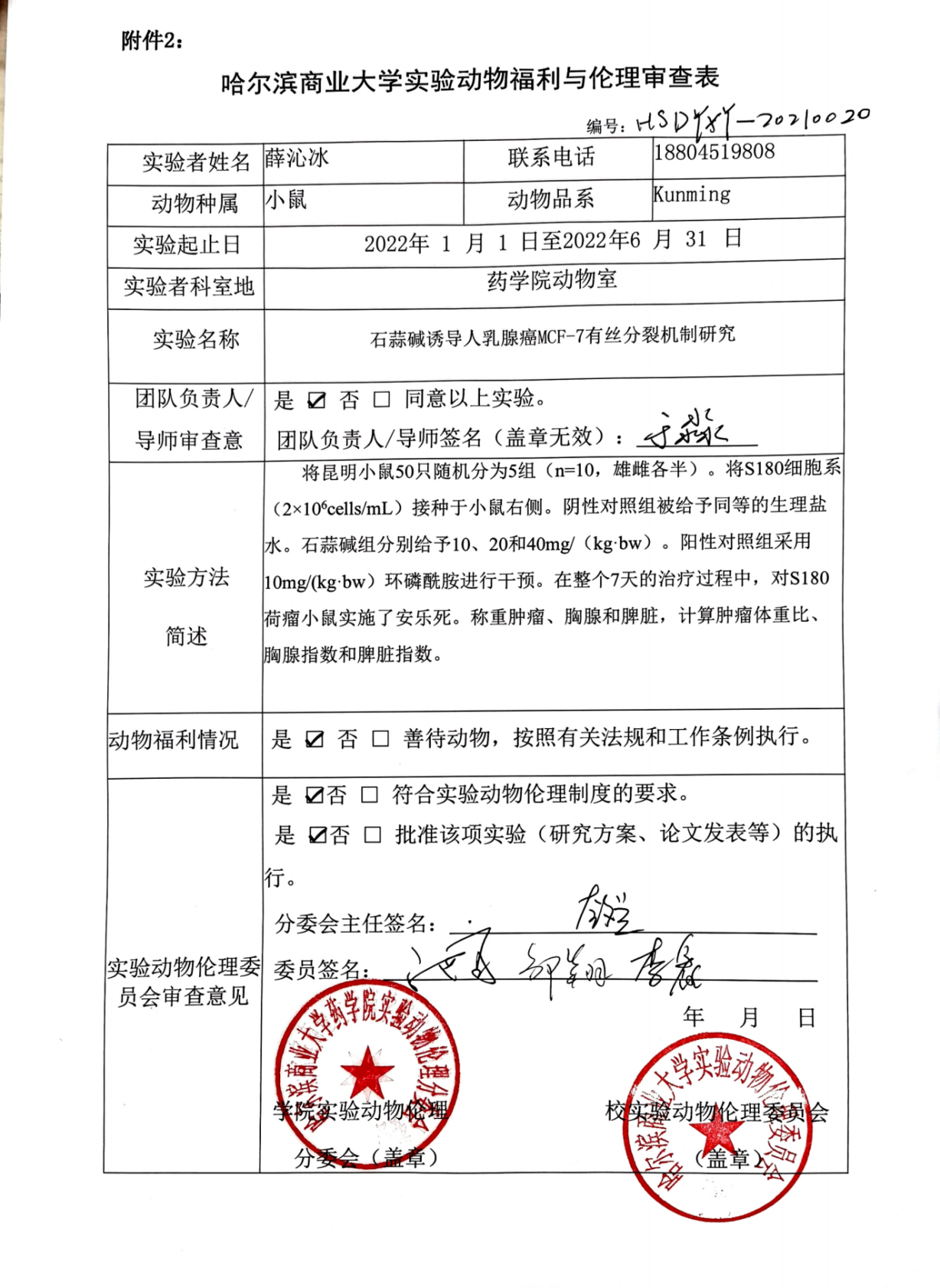


**Fig. S3** Affidavit of approval of animal ethics and welfare


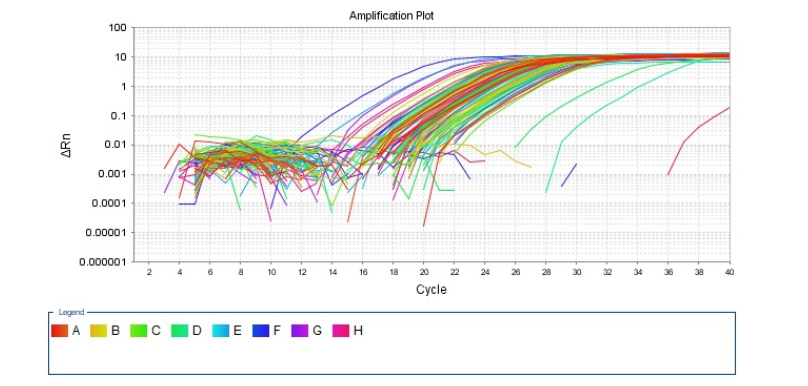


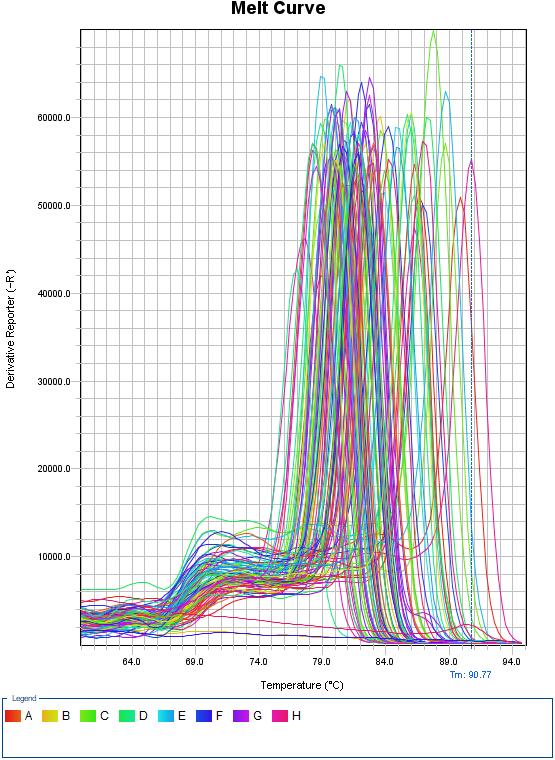


**Fig. S4** Primer sequences in PCR reaction system.


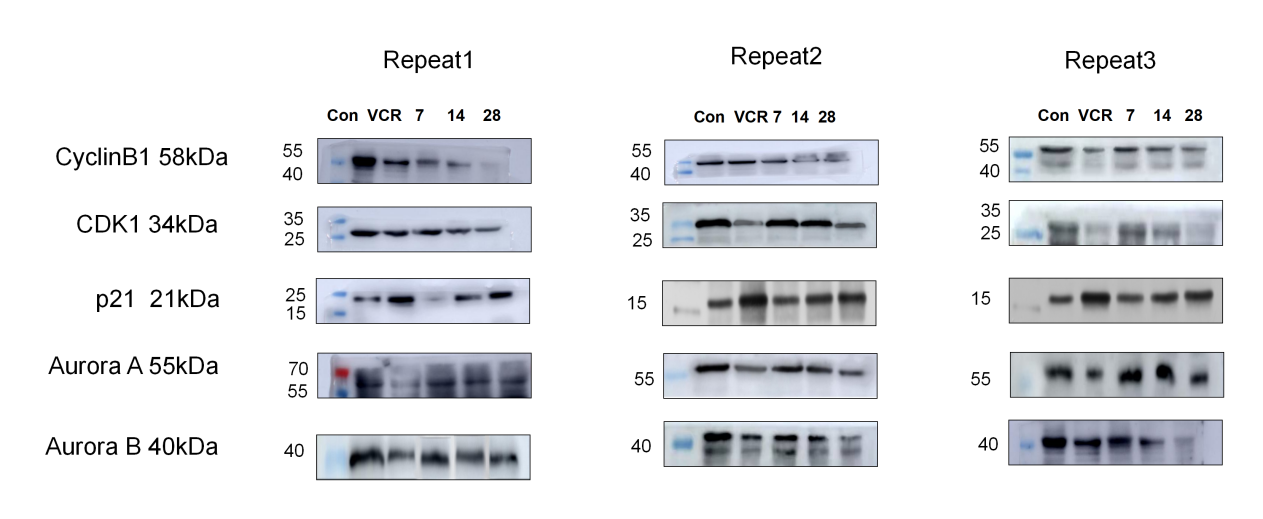


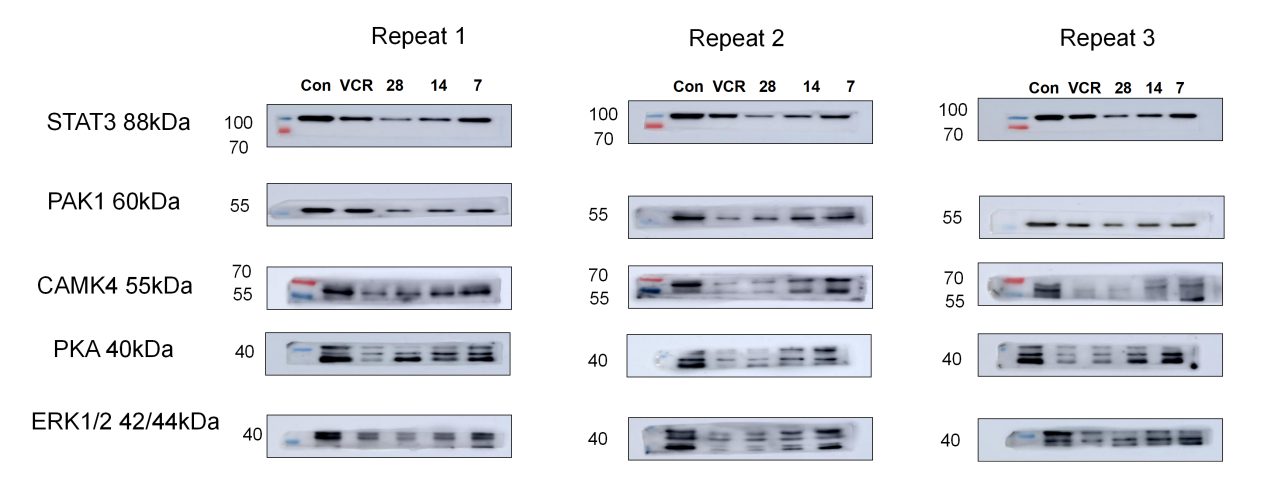


**Fig. S5** Effect of MCF-7 cell cycle arrest on protein expressions induced by lycorine (n=3)
